# Rett syndrome linked to defects in forming the MeCP2/Rbfox/LASR complex in mouse models

**DOI:** 10.1038/s41467-021-26084-3

**Published:** 2021-10-01

**Authors:** Yan Jiang, Xing Fu, Yuhan Zhang, Shen-Fei Wang, Hong Zhu, Wei-Kang Wang, Lin Zhang, Ping Wu, Catherine C. L. Wong, Jinsong Li, Jinbiao Ma, Ji-Song Guan, Ying Huang, Jingyi Hui

**Affiliations:** 1 State Key Laboratory of Molecular Biology, Center for Excellence in Molecular Cell Science, Shanghai Institute of Biochemistry and Cell Biology, Chinese Academy of Sciences, University of Chinese Academy of Sciences, 200031 Shanghai, China; 2 Shanghai Center for Plant Stress Biology, Chinese Academy of Sciences, 201602 Shanghai, China; 3 State Key Laboratory of Genetic Engineering, Collaborative Innovation Centre of Genetics and Development, Department of Biochemistry, Institute of Plant Biology, School of Life Sciences, Fudan University, 200438 Shanghai, China; 4 Department of General Surgery, Shanghai Key Laboratory of Biliary Tract Disease Research, State Key Laboratory of Oncogenes and Related Genes, Xinhua Hospital, Shanghai Jiao Tong University, 200092 Shanghai, China; 5 State Key Laboratory of Cell Biology, Shanghai Key Laboratory of Molecular Andrology, Shanghai Institute of Biochemistry and Cell Biology, Center for Excellence in Molecular Cell Science, Chinese Academy of Sciences, University of Chinese Academy of Sciences, 200031 Shanghai, China; 6 National Facility for Protein Science in Shanghai, Zhangjiang Lab, Shanghai Advanced Research Institute, Chinese Academy of Sciences, 201210 Shanghai, China; 7 School of Life Science and Technology, ShanghaiTech University, 201210 Shanghai, China; 8 Center for Excellence in Brain Science and Intelligence Technology, Chinese Academy of Sciences, 200031 Shanghai, China; 9 Present Address: Center for Precision Medicine Multi-Omics Research, Peking University Health Science Center, School of Basic Medical Sciences, Peking University, 100191 Beijing, China

**Keywords:** RNA-binding proteins, Alternative splicing

## Abstract

Rett syndrome (RTT) is a severe neurological disorder and a leading cause of intellectual disability in young females. RTT is mainly caused by mutations found in the X-linked gene encoding methyl-CpG binding protein 2 (MeCP2). Despite extensive studies, the molecular mechanism underlying RTT pathogenesis is still poorly understood. Here, we report MeCP2 as a key subunit of a higher-order multiunit protein complex Rbfox/LASR. Defective MeCP2 in RTT mouse models disrupts the assembly of the MeCP2/Rbfox/LASR complex, leading to reduced binding of Rbfox proteins to target pre-mRNAs and aberrant splicing of *Nrxns* and *Nlgn1* critical for synaptic plasticity. We further show that MeCP2 disease mutants display defective condensate properties and fail to promote phase-separated condensates with Rbfox proteins in vitro and in cultured cells. These data link an impaired function of MeCP2 with disease mutation in splicing control to its defective properties in mediating the higher-order assembly of the MeCP2/Rbfox/LASR complex.

## Abstract (web summary)

MeCP2 mutations can cause Rett syndrome, a severe childhood neurological disorder. Here the authors show that MeCP2 mediates the higher-order assembly of a large splicing complex Rbfox/LASR, which is disrupted in the mouse models of Rett syndrome.

## Introduction

Rett syndrome (RTT, OMIM #312750) is a postnatal neurological disorder and a leading cause of intellectual disability in girls. Individuals with RTT often display severe motor and cognitive impairments, respiratory and bone abnormalities, and autistic features^1,2^. Most cases with RTT are caused by mutations within the gene encoding for MeCP2 gene^3^, which is a member of the methyl-CpG-binding domain (MBD)-containing protein family that binds 5-methyl cytosine and 5-hydroxylmethyl cytosine residues across the entire genome^4–6^.

The MeCP2 protein contains an N-terminal domain (NTD), an MBD, an intervening domain (ID), a transcriptional repression domain (TRD), two AT-hook motifs, and a C-terminal domain (CTD). Common MeCP2 mutations seen in RTT are clustered within its MBD and TRD, and individuals with mutations in the MBD or large truncations often exhibit more severe symptoms than those with mutations outside MBD or small C-terminal truncations^1^. While ubiquitously expressed in a variety of tissues, MeCP2 is highly abundant in the brain at the level nearly comparable to that of histones^5^. Mouse models with the loss or mutations of MeCP2 either in the whole body or specifically in the brain recapitulate the cellular and behavioral phenotypes of RTT patients, which have been providing valuable animal models for studying RTT^7,8^. However, despite significant progresses over the past 2–3 decades, the molecular mechanism underlying the pathogenesis of RTT caused by MeCP2 dysfunction is still poorly understood.

MeCP2 appears to act in a multifaceted manner in regulating gene expression. As a protein binding to methylated DNA, MeCP2 has been implicated in transcriptional repression or activation through binding to transcription co-repressor complexes or transcription activator^9–12^. Additionally, MeCP2 has also been suggested to play a role in alternative pre-mRNA splicing, another fundamental process during gene expression^13,14^. The initial link between MeCP2 and splicing regulation was identified by showing that MeCP2 interacts with the splicing regulator YB-1 in an RNA-dependent manner and modulates the splicing of a minigene reporter for an YB-1 target^15^. Later, Li et al. identified hundreds of altered splicing events in MeCP2 knockout (KO) mice by RNA sequencing (RNA-seq) and proposed that MeCP2 regulates the splicing of α-amino-3-hydroxy-5-methyl-4-isoxazolepropionic acid receptor (AMPAR) genes through recruiting LEDGF^16^. However, the mechanism underlying MeCP2-mediated splicing regulation in RTT has remained largely elusive.

In the brain, the RNA-binding fox-1 (Rbfox) family, consisting of Rbfox1 (A2BP1), Rbfox2 (RBM9), and Rbfox3 (NeuN), has been well established to play critical roles in splicing regulation. Consequently, conditional KO of *Rbfox1* in mouse central nervous system (CNS) causes susceptibility to seizures and neuronal hyperexcitation^17^, and Rbfox1 in interneuron is required for the establishment of connectivity within related classes of interneurons^18^. CNS- or neural crest cell-specific *Rbfox2* KO mice displayed defects in cerebellar development and craniofacial bone development, respectively^19,20^. Rbfox1/2/3 triple KO in an in vitro neuronal maturation system exhibited immature electrophysiological activity and defects in the assembly of axon initial segment^21^. The Rbfox proteins contain a single typical RNA recognition motif domain that recognizes the (U)GCAUG motif with high affinity^22,23^. Mechanistically, they tend to bind intron regions downstream of alternative exons to activate splicing and bind alternative exons or their upstream regions to inhibit splicing^24,25^. Recently, the Rbfox proteins have been shown to form a multi-subunit protein complex in cultured cells and mouse brain, termed large assembly of splicing regulators (LASR). Besides Rbfox proteins, the LASR complex harbors multiple other RNA binding proteins, including hnRNP M, hnRNP H, hnRNP C, hnRNP U, Matrin3, NF45/110, and DDX5^26^. Rbfox proteins mainly associate with LASR in the nucleus and can regulate splicing through interaction networks within LASR complex^26^. Interestingly, the CTD of Rbfox proteins has been shown to mediate the higher-order assembly of this complex, which proves to be essential for its splicing regulatory activity^27^. Despite their broad contribution to neurological disorders in the brain, Rbfox proteins have not yet been functionally connected to RTT.

In this work, we identify MeCP2 as a key subunit of the Rbfox/LASR complex. Depletion of MeCP2 disrupts the formation of the Rbfox/LASR complex in cultured cells and results in decreased binding of RBFOX2 to the Rbfox/LASR pre-mRNA targets. In MeCP2 mutant mice, defects in forming the Rbfox/LASR complex lead to aberrant splicing of genes critical for synaptic transmission. These functions are further correlated to the ability of MeCP2 to form nuclear condensates with Rbfox proteins, a biochemical property abrogated by RTT-causing mutations in MeCP2. Taken together, these results reveal a mechanism that may underlie RTT pathogenesis where MeCP2 functions in splicing control through driving the assembly of the Rbfox/LASR complex.

## Results

### MeCP2 is a new subunit of the Rbfox/LASR complex

To understand the molecular function of MeCP2 in gene expression, we first searched for MeCP2 interacting partners using immunoprecipitation coupled with mass spectrometry (IP-MS) analysis. Given that MeCP2 is a chromatin-bound nuclear protein and that insoluble chromatin pellet is difficult for IP, we adopted a protocol similar to a previously described method^26^ to prepare subnuclear extract containing high-molecular-weight (HMW) material, which was purified under low salt condition to preserve complexes formed by protein–protein interactions upon release from chromatin pellet by nuclease treatment (Supplementary Fig. 1a). Both FLAG-tagged MeCP2 expressed in HEK293T cells and endogenous MeCP2 extracted from mouse brain were enriched in the HMW extract (Supplementary Fig. 1b, c).

**Fig. 1:**
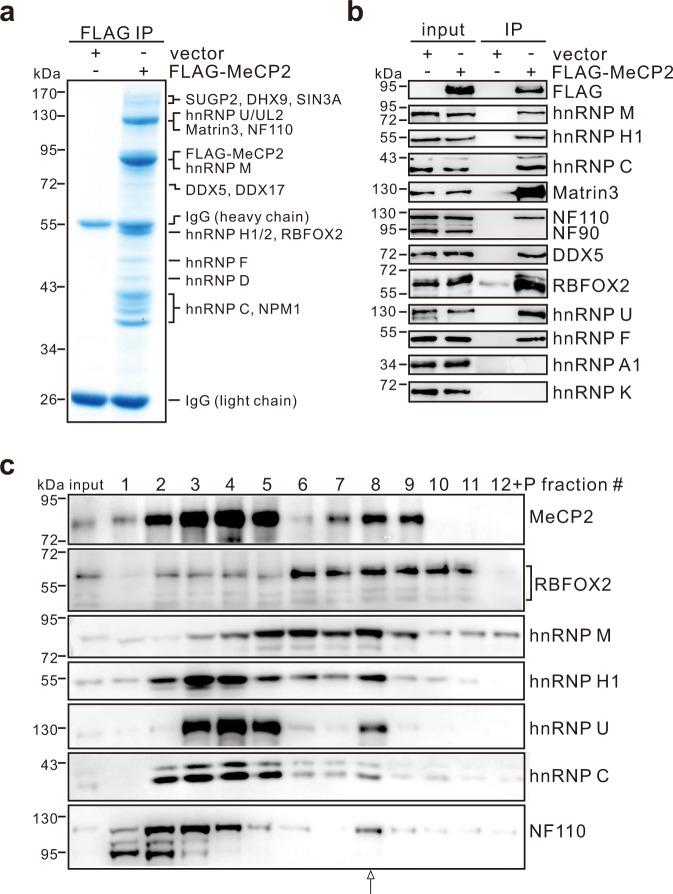
MeCP2 is a new subunit of Rbfox/LASR complex. **a** Coomassie blue staining image of SDS-PAGE gel showing proteins IPed by anti-FLAG antibody from the HMW extract of HEK293T cells expressing FLAG-MeCP2. Proteins determined by mass spectrometry are indicated on the right. **b** IP assays performed with anti-FLAG antibody and analyzed by western blotting with the indicated antibodies. **c** Sedimentation profiles of MeCP2, RBFOX2, and LASR components in the HMW extract from HEK293T cells on 10–50% glycerol gradients. Fractions #1–12 were collected from top to bottom. Source data are provided as a Source data file.

We immunopurified FLAG-MeCP2 interacting proteins from the HMW extract of HEK293T cells and determined the identity of specific co-purifying proteins on Coomassie blue-stained gel by MS (Fig. 1a). Remarkably, MeCP2 co-precipitated with hnRNP U/UL2, Matrin3, NF110, hnRNP M, DDX5/17, hnRNP H1/2, and hnRNP C, as well as RBFOX2, which are major components of the higher-order Rbfox/LASR complex recently elucidated and characterized^26^. RBFOX2 is the only member of Rbfox protein family expressed in HEK293T cells. The protein–protein interactions between MeCP2 and components of the RBFOX2/LASR complex after nucleic acid digestion were confirmed by western blot analysis (Fig. 1b). As a negative control, MeCP2 showed no interactions with NF90, hnRNP A1, and hnRNP K, which are absent in LASR complex, indicating that the interactions between MeCP2 and components of RBFOX2/LASR are specific. To investigate whether MeCP2 associates with RBFOX2/LASR as a whole complex, the HMW extracts were fractionated on 10–50% glycerol gradients (Fig. 1c), revealing that both MeCP2 and RBFOX2/LASR constituent proteins co-sediment on a peak at fraction #8. We also noticed that portions of MeCP2 sedimented as smaller complexes to the lower-molecular-weight fractions #2–5. These results indicate that MeCP2 is a subunit of the previously identified RBFOX2/LASR complex.

### MeCP2 directly interacts with RBFOX2 via its MBD and ID

**Fig. 2:**
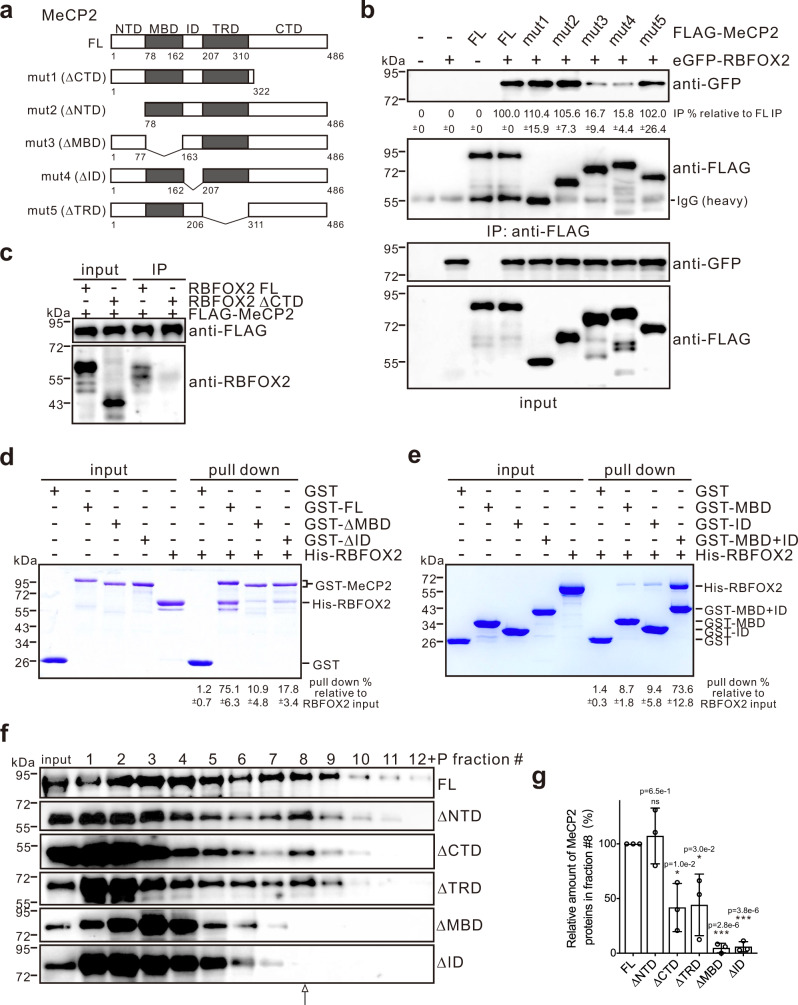
MeCP2 interacts with RBFOX2 directly, and its MBD and ID are required for its association with RBFOX2/LASR complex. **a** Schematic representation of FL and deletion mutants of MeCP2. **b** Co-IP assays performed by anti-FLAG antibody in HEK293T cells expressing FLAG-tagged FL or deletion mutants of MeCP2 in the presence of nuclease and analyzed by western blotting with anti-GFP antibody. The average percentages of IP efficiency with standard deviations relative to the FL MeCP2 IP efficiency are shown below the gel (*n* = 3 biologically independent experiments). **c** IP assays performed by anti-FLAG antibody in HEK293T cells expressing RBFOX2 FL or its ΔCTD mutant in the presence of nuclease and analyzed by western blotting with anti-RBFOX2 antibody. **d** Representative results for GST pull-down analysis of the interactions between GST-tagged FL, ΔMBD, and ΔID of MeCP2 proteins and His-tagged RBFOX2. **e** Representative results for GST pull-down analysis of the interactions between GST-tagged MBD, ID, and MBD + ID of MeCP2 proteins and His-tagged RBFOX2. The average percentages of pulled down RBFOX2 relative to the input with standard deviations are shown below the gel in **d**, **e** (*n* = 3 biologically independent experiments). **f** Representative results for sedimentation profiles of the FL and mutant MeCP2 proteins in the HMW extract from HEK293T cells expressing FLAG-tagged MeCP2 proteins on 10–50% glycerol gradients. The FL and mutant MeCP2 proteins were detected by western blot analysis with anti-FLAG antibody. **g** Quantitation of the relative amount of MeCP2 proteins associated with Rbfox/LASR. The average percentages of MeCP2 proteins in fraction #8 to the sum of those in total fractions are normalized to that of FL MeCP2. Error bars represent standard deviation (*n* = 3 biologically independent experiments, *p < 0.05, ***p < 0.001, two-sided Student’s *t* test). Source data are provided as a Source data file.

To understand how MeCP2 associates with the RBFOX2/LASR complex, we first examined the interaction by co-IP between MeCP2 and a key member of the Rbfox/LASR complex, RBFOX2. Compared to full-length (FL) MeCP2, deletion of the NTD, CTD, or TRD showed little impact on their interactions with RBFOX2, and in contrast, mutant MeCP2 missing the MBD or ID significantly decreased the interactions (Fig. 2a, b). Similar to the interaction between Rbfox proteins and LASR components as reported earlier^27^, the interaction with MeCP2 also requires the CTD of RBFOX2 (Fig. 2c). Moreover, we performed a glutathione *S*-transferase (GST) pull-down assay and found that the recombinant GST-MeCP2 fusion protein was able to directly interact with His-tagged RBFOX2 in vitro, and the MBD and ID of MeCP2 were necessary and sufficient for such interaction (Fig. 2d, e).

Next, to determine which region of MeCP2 is important for forming a higher-order assembly with RBFOX2/LASR, we prepared HMW extracts from cells expressing FLAG-tagged FL or mutant MeCP2 proteins. Comparing to FL MeCP2, a similar sedimentation profile with the RBFOX2/LASR was observed with MeCP2 lacking the NTD, reduced with MeCP2 missing the CTD or TRD, and most importantly, MeCP2 without the MBD or ID failed to co-sediment with RBFOX2/LASR (Fig. 2f, g). Collectively, these data show that the association between MeCP2 and RBFOX2/LASR largely depends on MBD and ID with certain contribution from CTD and TRD.

### Loss of MeCP2 disrupts RBFOX2/LASR assembly

**Fig. 3:**
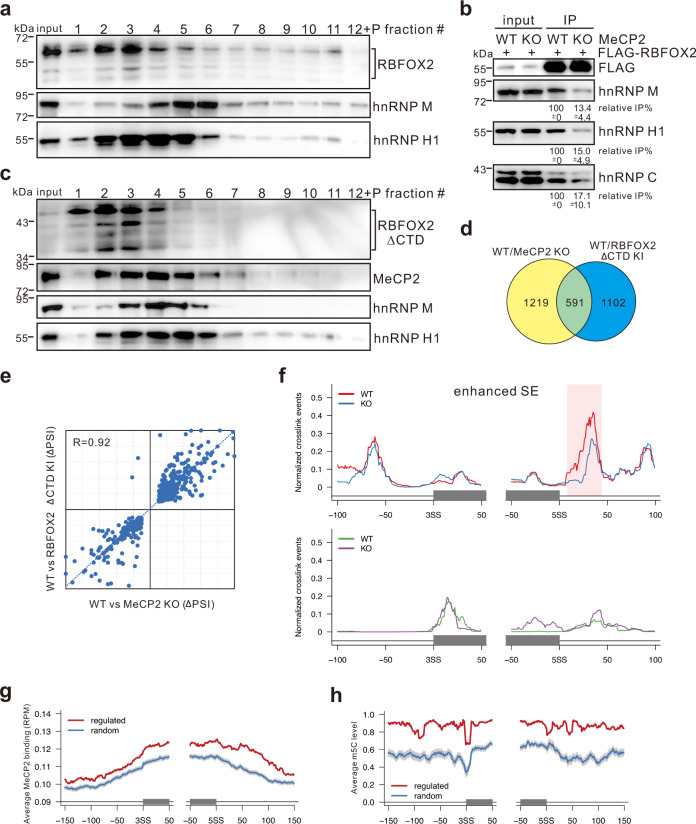
Loss of MeCP2 results in the dissociation of Rbfox/LASR complex and dysregulation of alternative splicing. **a** Sedimentation profiles of RBFOX2, hnRNP M, and hnRNP H1 in HMW extract from MeCP2 KO HEK293T cells on 10–50% glycerol gradients. **b** FLAG-IP assays performed in WT or MeCP2 KO cells expressing FLAG-tagged RBFOX2 and analyzed by western blot with the indicated antibodies. The average percentages of RBFOX2 IP efficiency with standard deviations in MeCP2 KO cells relative to WT cells are shown below the gel (*n* = 3 biologically independent experiments). **c** Sedimentation profiles of RBFOX2 ΔCTD, MeCP2, hnRNP M, and hnRNP H1 in HMW extract from WT or RBFOX2 KI HEK293T cells on 10–50% glycerol gradients. **d** Venn chart depicting overlapped splicing events between WT vs MeCP2 KO and WT vs RBFOX2 KI. **e** Scatter plot showing splicing change ΔPSI (percent spliced in) values for overlapped splicing events between WT vs MeCP2 KO and WT vs RBFOX2 KI. **f** RNA map showing in vivo RBFOX2 binding to pre-mRNAs with skipped exon (SE) splicing pattern regulated by both MeCP2 and RBFOX2/LASR in WT (red or green) or MeCP2 KO (blue or purple) cells determined by iCLIP-seq. Gray boxes represent the alternative exons stimulated (upper) or repressed (lower) by both MeCP2 and RBFOX2/LASR. The red box indicates the region bound by RBFOX2 with significant difference between WT and MeCP2 KO cells (Bonferroni adjusted *p* value < 0.01 and fold change >2). **g**, **h** MeCP2 binding (**g**) and m5C levels (**h**) of genomic regions for MeCP2 and RBFOX2/LASR co-regulated splicing events determined by MeCP2 ChIP-seq or WGBS analyses. The red line represents the mean values calculated for the co-regulated events, and the blue line for randomly selected events. The gray shadow represents a 99% confidence interval (CI). Source data are provided as a Source data file.

To determine whether the loss of MeCP2 has any effects on the integrity of the RBFOX2/LASR complex, we depleted MeCP2 in HEK293T cells using CRISPR/Cas9 gene editing technology. The expression levels of RBFOX2 and LASR components did not show significant changes upon MeCP2 depletion (Supplementary Fig. 2). However, on glycerol gradients, RBFOX2 and representative LASR components hnRNP M and hnRNP H1 all sedimented to fractions with lower molecular weight in MeCP2 KO cells compared to wild-type (WT) cells (Fig. 3a). Consistently, RBFOX2 showed reduced interaction with LASR components in MeCP2 KO cells relative to WT cells (Fig. 3b). These data demonstrate that MeCP2 is essential for the assembly of the RBFOX2/LASR complex in HEK293T cells.

### Dissociated RBFOX2/LASR leads to splicing changes in MeCP2 KO cells

Since Rbfox CTD-mediated interaction with LASR is required for the higher-order formation of Rbfox/LASR^27^, we wondered whether the association of MeCP2 with Rbfox/LASR is disrupted in the absence of Rbfox CTD. We thus generated a stable cell line expressing RBFOX2 without CTD (RBFOX2 ΔCTD KI) using CRISPR/Cas9 knock-in (KI) technology (Supplementary Fig. 2). These cells express a truncated RBFOX2 protein with an SV40 nuclear localization signal (NLS) introduced at its C-terminus. In RBFOX2 ΔCTD KI cells, LASR components and MeCP2 failed to co-sediment in the corresponding fraction #8 on glycerol gradients (Fig. 3c), indicating that the KI cell line mimicked the destruction of RBFOX2/LASR complex.

Next, we performed RNA-seq analysis with total RNA isolated from WT, MeCP2 KO, and RBFOX2 KI cells. In total, we detected 1810 significantly changed splicing events for WT vs MeCP2 KO cells and 1693 splicing events for WT vs RBFOX2 KI cells. Among these, 591 splicing events were commonly induced by MeCP2 KO and RBFOX2 ΔCTD KI (Fig. 3d and Supplementary Data 1), displaying a positive correlation (Fig. 3e). In contrast to LASR components that also exist as smaller subcomplexes, the majority of nuclear Rbfox proteins associate with LASR complex and interact with introns^26^. We therefore monitored changes in RBFOX2 binding to RNA between WT and MeCP2 KO cells by performing RBFOX2 individual nucleotide resolution ultraviolet (UV) cross-linking and immunoprecipitation sequencing (iCLIP-seq; Supplementary Data 2). Compared to WT cells, loss of MeCP2 clearly led to a decrease in RBFOX2 binding to intronic regions downstream of the alternatively spliced exons co-activated by MeCP2 and RBFOX2/LASR (Fig. 3f). These data indicate that disrupting the integrity of the RBFOX2/LASR complex is responsible for a significant fraction of splicing changes induced by the loss of MeCP2 and decreased binding of RBFOX2 to its pre-mRNA targets.

Since MeCP2 is a methylated DNA-binding protein, we further investigated whether MeCP2 binding and DNA methylation are involved in the regulation of splicing targets for MeCP2/Rbfox/LASR. MeCP2 chromatin immunoprecipitation–sequencing (ChIP-seq) analysis revealed that the genomic regions for MeCP2/RBFOX2/LASR regulated exons and their flanking sequences bound more MeCP2 than those regions from randomly selected events (Fig. 3g). Using a public dataset of 5-methylcytosine (m5C) methylome in HEK293 cells determined by whole-genome bisulfite sequencing (WGBS)^28^, we found that the m5C methylation level is higher over the regulated alternative exons and their flanking regions than the regions associated with randomly selected splicing events (Fig. 3h), suggesting that MeCP2 acts as a bridge between methylated DNA and RBFOX2/LASR on their regulated pre-mRNA targets. This is illustrated on a known target for Rbfox, the exon EIIIB of *FN1*^29^ (Supplementary Fig. 3). Combining the results shown in Fig. 2, we hypothesized that the MBD and ID of MeCP2 are important for its “bridge” role. We performed pull-down assays and found that the biotinylated DNA probe containing m5C was able to interact with RBFOX2 through the MBD and ID of MeCP2 (Supplementary Fig. 4).

To gain further insights into the features of the MeCP2/Rbfox/LASR complex, we treated HEK293T cells with a DNA methyltransferase inhibitor 5-aza-2′-deoxycytidine (5-Aza) or a transcription inhibitor 5,6-dichloro-1-β-D-ribofuranosylbenzimidazole (DRB). Both treatments resulted in the dissociation of the MeCP2/Rbfox/LASR complex, while it was restored after DRB washout (Supplementary Fig. 5a, b). These data are consistent with the possibility that the higher-order MeCP2/Rbfox/LASR complex is co-transcriptionally assembled on chromatin, which depends on both DNA methylation and nascent RNA synthesis.

### Compromised Rbfox/LASR complex in RTT mice

**Fig. 4:**
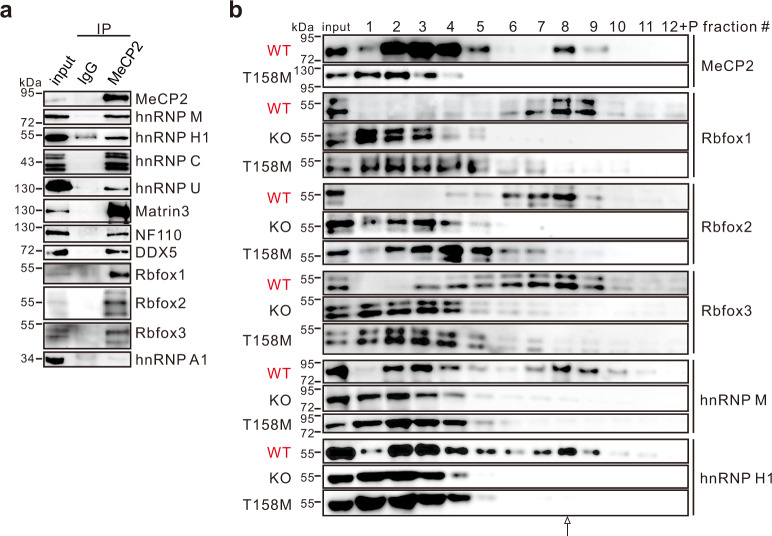
Disruption of Rbfox/LASR complex in MeCP2 KO and T158M mice. **a** IP assays were performed using anti-MeCP2 antibody and analyzed by western blotting using the HMW extract from the mouse cerebral cortex. **b** Sedimentation profiles of MeCP2, Rbfox proteins, and LASR components in the HMW extract from the cerebral cortex of MeCP2 WT, KO, and T158M mice on 10–50% glycerol gradients. Source data are provided as a Source data file.

Next, we investigated the formation of the MeCP2/Rbfox/LASR complex in mice through IP experiments using HMW extract prepared from mouse cerebral cortex. We observed that MeCP2 efficiently co-IPed with all three Rbfox proteins and all the key components of LASR tested (Fig. 4a). Importantly, compared to WT mice, Rbfox proteins and LASR components failed to co-sediment to the corresponding fraction containing the MeCP2/Rbfox/LASR complex prepared from cerebral cortex or cerebellum of MeCP2^−/y^ and MeCP2^T158M/y^ mice (Fig. 4b and Supplementary Fig. 6), as similarly observed in MeCP2 KO HEK293T cells (Fig. 3a). The T158M mutation is located in the MBD domain and represents one of the most frequent mutations found in RTT patients, which has been demonstrated to impair the DNA binding and stability of MeCP2^30,31^. Together, these data indicate that MeCP2 acts as a scaffold for the assembly of the Rbfox/LASR complex in both cultured cells and mouse brain.

### Defective MeCP2/Rbfox/LASR causes aberrant splicing in RTT mice

In an attempt to search for critical targets of MeCP2/Rbfox/LASR, we performed RNA-seq on total RNA isolated from cerebral cortex of MeCP2^+/y^, MeCP2^−/y^, or MeCP2^T158M/y^ mice. No apparent changes in mRNA for Rbfox proteins and key LASR components were detectable between MeCP2 mutant and WT mice by RNA-seq, also confirmed at protein levels by western blot analyses (Supplementary Fig. 7a, b). The RNA-seq analysis identified 751 splicing changes between WT and MeCP2 KO mice and 689 splicing changes between WT and MeCP2 T158M mice (Supplementary Data 3 and 4). Forty-seven out of 384 cassette exon type of splicing changes induced by MeCP2 KO and 38 out of 380 events induced by MeCP2 T158M mutation were previously predicted as targets for Rbfox proteins based on integrative modeling using a Bayesian Network^29^.

**Fig. 5:**
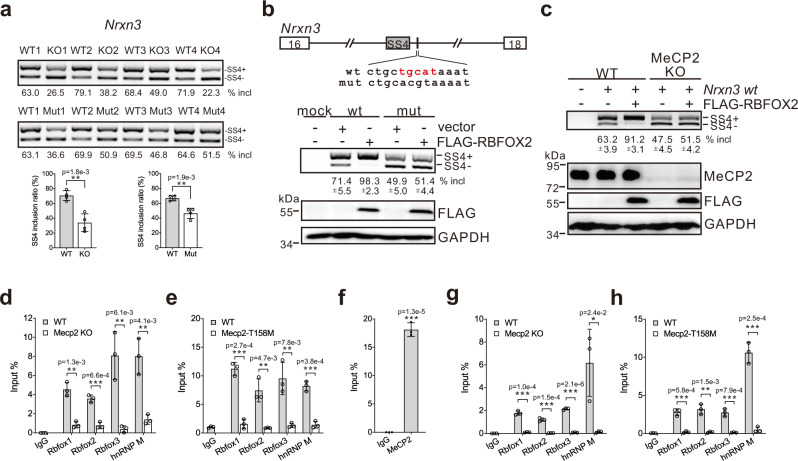
Regulation of *Nrxn3* exon SS4 splicing by Rbfox/LASR in MeCP2 KO and T158M mice. **a** Alternative splicing of *Nrxn3* in the cerebral cortex of MeCP2 WT, KO, and T158M detected by RT-PCR. The average percentages of exon inclusion with standard deviations are shown below the gel images (*n* = 4 biological replicates; **p < 0.01, two-sided Student’s *t* test). **b** Schematic representation of *Nrxn3* minigenes and RT-PCR analysis of in vivo splicing of *Nrxn3* minigenes (wt and mut) in HEK293T cells transfected with a vector or a FLAG-RBFOX2 expression construct. The average percentages of exon inclusion with standard deviations are shown below (*n* = 3 biologically independent experiments). **c** RT-PCR analysis of in vivo splicing of *Nrxn3* wt minigene in WT or MeCP2 KO HEK293T cells transfected with a vector or a FLAG-RBFOX2 expression construct. The average percentages of exon inclusion with standard deviations are shown below (*n* = 3 biologically independent experiments). **d**, **e** CLIP-RT-qPCR analyses of Rbfox proteins and hnRNP M binding to *Nrxn3* pre-mRNAs in MeCP2 KO (**d**) or T158M (**e**) mice compared to WT mice. **f** ChIP-qPCR analysis of MeCP2 binding to *Nrxn3* genomic locus in WT mice. **g**, **h** ChIP**-**qPCR analyses of Rbfox proteins and hnRNP M associating with *Nrxn3* genomic locus in MeCP2 KO (**g**) or T158M (**h**) mice compared to WT mice. Error bars in **d**–**h** represent standard deviations (*n* = 3 biologically independent experiments; *p < 0.05, **p < 0.01, ***p < 0.001, two-sided Student’s *t* test). Source data are provided as a Source data file.

Since both MeCP2 KO and T158M mouse models showed defects in forming the Rbfox/LASR complex, we hypothesized that some of the overlapped splicing changes between the two mouse models might be targets for Rbfox/LASR with critical biological functions. Eighty-four overlapped splicing events in both mouse models were detected, which are enriched in the Gene Ontology (GO) terms associated with synaptic plasticity (Supplementary Fig. 8a–d and Supplementary Data 5). The relatively small number of overlapped splicing targets is probably due to the different disease processes between the two RTT models. Among these overlapped targets, we found Rbfox-binding sites located closely to some regulated alternative exons, and 20 out of 69 overlapped cassette exons are candidate targets for Rbfox proteins as predicted^29^. Among these are Neurexins (Nrxn1, Nrxn2, and Nrxn3) and Neuroligin 1 (Nlgn1), both being transmembrane cell adhesion proteins important for synaptic function^32^. We found that the inclusion of exon SS4 in all three Neurexin homologs and the inclusion of *Nlgn1* exon 4 are downregulated in both mouse models (Fig. 5a and Supplementary Fig. 8b–d).

To further characterize these targets, we constructed a *Nrxn3* minigene and mutated a potential Rbfox-binding site downstream of exon SS4. Reverse transcriptase polymerase chain reaction (RT-PCR) analyses of in vivo splicing assay showed that RBFOX2 promoted the inclusion of *Nrxn3* exon SS4 in WT but not in mutant minigene through this binding site (Fig. 5b). Depletion of MeCP2 resulted in reduced inclusion of *Nrxn3* exon SS4, and RBFOX2 was able to enhance exon SS4 splicing in WT but not in MeCP2 KO cells (Fig. 5c), indicating that exon SS4 inclusion stimulated by RBFOX2 is dependent on MeCP2. Moreover, we found that *Nlgn1* exon 4 inclusion repressed in two MeCP2 mutant mouse models (Supplementary Fig. 8b–d) was also regulated by RBFOX2 through an Rbfox-binding motif downstream of exon 4 (Supplementary Fig. 9a).

Corroborating with these findings in transfected cells, we detected no obvious changes at the total mRNA levels of *Nrxns* and *Nlgn1* between WT and MeCP2 mutant mice (Supplementary Fig. 10a, b). However, CLIP-RT-qPCR analysis showed that Rbfox proteins exhibited decreased binding to *Nrxn3* or *Nlgn1* pre-mRNAs in MeCP2 KO or T158M mice than in WT mice (Fig. 5d, e and Supplementary Fig. 9b, c). Moreover, ChIP-qPCR results showed that MeCP2 bound *Nrxn3* or *Nlgn1* genomic locus (Fig. 5f and Supplementary Fig. 9d) and that the association of Rbfox proteins with *Nrxn3* or *Nlgn1* genomic DNA locus reduced in MeCP2 KO or T158M mice compared to that in WT mice (Fig. 5g, h and Supplementary Fig. 9e, f). Meanwhile, LASR component, hnRNP M, also bound less to the pre-mRNA and genomic locus of *Nrxn3* or *Nlgn1* in MeCP2 mutant mice (Fig. 5d, e, g, h and Supplementary Fig. 9b, c, e, f). Taken together, these data strongly suggest that the MeCP2/Rbfox/LASR complex helps to hold its components at a specific micro-compartment with a relatively high local concentration. Defects in this process led to the reduced binding of Rbfox proteins and hnRNP M to their target gene loci and pre-mRNAs, in turn changing splicing of those genes important for neurotransmission, which may contribute to RTT.

### MeCP2 forms liquid-like condensates both in vitro and in vivo

We next explored the puzzle why MeCP2 is essential for the formation of MeCP2/Rbfox/LASR complex. Recent studies suggest that proteins with intrinsically disordered regions (IDRs) or low-complexity regions (LCRs) form biomolecular condensates through liquid–liquid phase separation (LLPS)^33^. Given that 60% of MeCP2 is composed of IDRs distributed throughout the entire protein^34^, we hypothesized that MeCP2 may possess LLPS property to fulfill its scaffolding function. To test this hypothesis, we first purified recombinant FL MeCP2 fused to enhanced green fluorescence protein (eGFP) from bacteria. By confocal microscopy, spherical droplets of MeCP2 became larger with increasing concentration (Supplementary Fig. 11a), which could fuse into larger droplets (Supplementary Fig. 11b). Fluorescence recovery after photobleaching (FRAP) showed recovery of MeCP2 droplets after photobleaching (Supplementary Fig. 11c, d). In contrast to WT protein, MeCP2 mutants R106W and T158M formed less and smaller droplets (Supplementary Fig. 11e). MeCP2 R106W is another common mutation found in the MBD domain, which has a more severe phenotype than T158M^35^. To investigate whether MeCP2 undergoes phase separation in vivo, we used CRISPR/Cas9 technology to generate an HEK293T KI cell line expressing eGFP-tagged MeCP2 protein driven by its endogenous promoter. MeCP2-eGFP fusion proteins formed nuclear puncta, which were able to undergo fusion or fission rapidly (Supplementary Fig. 11f–h). Collectively, our data demonstrate that MeCP2 has a physical property of LLPS both in vitro and in vivo.

### MeCP2, but not its disease mutant, forms condensates with Rbfox proteins

**Fig. 6:**
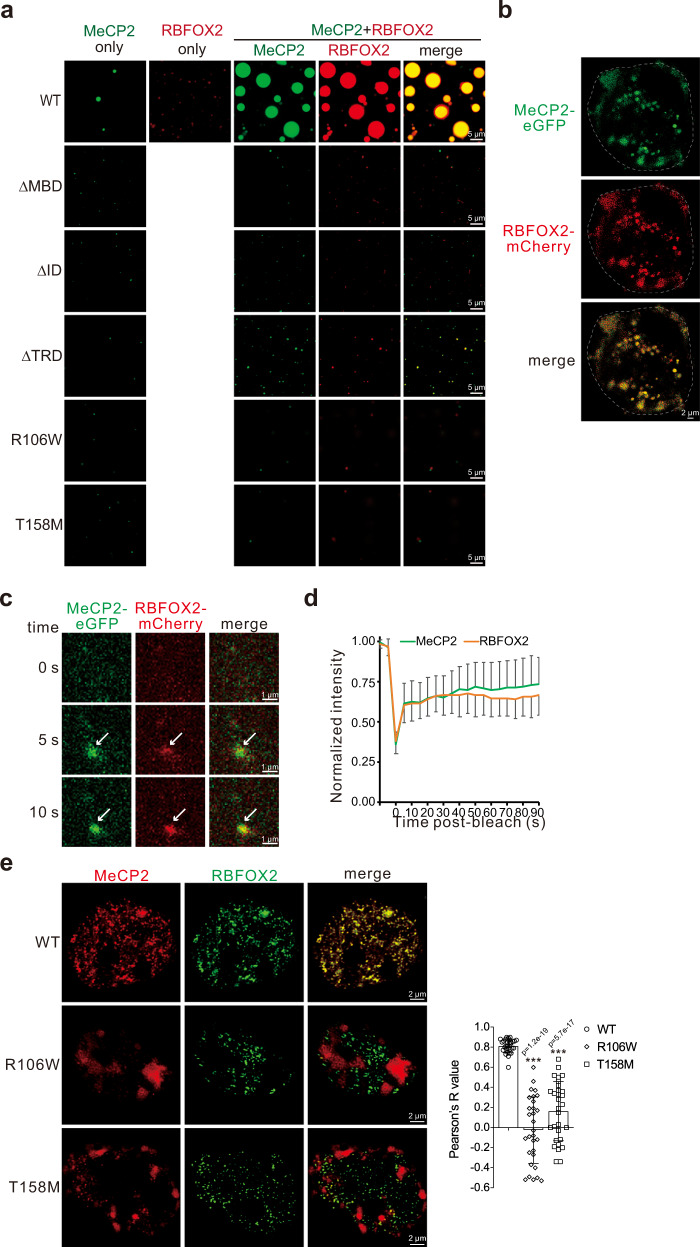
WT MeCP2, but not its disease mutants, forms liquid-like condensates with RBFOX2. **a** Representative fluorescence images of droplets formed by MeCP2 WT, ΔMBD, ΔID, ΔTRD, R106W, or T158M alone or together with RBFOX2 at 150 mM salt condition. Scale bar, 5 µm. **b** Representative live cell images of eGFP-tagged MeCP2 and mCherry-tagged RBFOX2 in HEK293T knock-in cells. The nucleus is outlined by dotted lines. The average Pearson’s *R* value with a standard deviation is 0.48 ± 0.13 (*n* = 15 cells). Scale bar, 2 µm. **c** The dynamics of condensate formation by MeCP2-eGFP and RBFOX2-mCherry in HEK293T knock-in live cells. Scale bar, 1 µm. **d** Quantitation of FRAP analysis of puncta with both MeCP2-eGFP and RBFOX2-mCherry in HEK293 knock-in cells. Error bars represent standard deviations (*n* = 20 cells). **e** Representative confocal fluorescence microscopic images of RBFOX2, WT, and mutant MeCP2 proteins in HEK293T live cells. Pearson’s *R* value analyses are shown on the right. Error bars represent standard deviations (*n* = 30 cells; ***p < 0.001, two-sided Student’s *t* test). Scale bar, 2 µm. Source data are provided as a Source data file.

Rbfox proteins have been previously shown to self-aggregate through an LCR within the CTD^27^. To test whether MeCP2 and RBFOX2 undergo co-aggregation, we performed in vitro droplet formation assays using purified MeCP2 and RBFOX2. Under the physiological salt condition, WT MeCP2 formed smaller spherical droplets, while the droplets formed by RBFOX2 were almost invisible (Fig. 6a). Strikingly, upon mixing, MeCP2 co-phase separated with RBFOX2, forming many larger droplets, while MeCP2 ΔMBD, ΔID, and ΔTRD mutants failed to do so, although the ΔTRD and RBFOX2 formed somewhat smaller droplets (Fig. 6a). Notably, MeCP2 disease mutants R106W and T158M, which showed impaired phase separation, also failed to co-phase separate with RBFOX2 (Fig. 6a). As a non-interacting control for MeCP2, MeCP2 did not co-phase separate with hnRNP A1 (Supplementary Fig. 12). These data indicate that WT MeCP2, but not MeCP2 with disease mutations, co-phase separated with RBFOX2 in vitro.

To investigate whether MeCP2 and RBFOX2 form condensates in cells, we further determined the cellular localization of MeCP2 and RBFOX2 and observed that endogenously eGFP-tagged MeCP2 and mCherry-tagged RBFOX2 were partially co-localized in HEK293T live cells (Fig. 6b). The nuclear condensates containing both MeCP2-eGFP and RBFOX2-mCherry proteins formed quickly and displayed dynamic internal rearrangement and rapid exchange kinetics in the FRAP assay (Fig. 6c, d). Next, we overexpressed MeCP2 and RBFOX2 proteins in HEK293T cells and monitored the dynamic localization/association of MeCP2 mutants and RBFOX2 in live cells. Similar to endogenous proteins, a significant fraction of WT MeCP2 and RBFOX2 co-localized within the punctate structure, while MeCP2 R106W and T158M mutant proteins were unevenly distributed in the nuclei and formed large clusters that were not co-localized with RBFOX2 (Fig. 6e). Results from FRAP assays showed that MeCP2 and RBFOX2 formed condensates with liquid-like property in HEK293T live cells (Supplementary Fig. 13a, b). In MeCP2 KO live cells, the puncta formed by RBFOX2 exhibited much less liquid-like property, and co-expression of WT MeCP2, but not R106W or T158M, rescued the dynamic exchange of RBFOX2 (Supplementary Fig. 13c, d). In addition, treatment of HEK293T cells with 5-Aza resulted in fewer puncta containing MeCP2 and RBFOX2 (Supplementary Fig. 14a). DRB treatment showed a similar effect, while the puncta containing both proteins were re-formed after DRB washout (Supplementary Fig. 14b). These data indicate that MeCP2 and RBFOX2 form liquid-like condensates in cultured cells, which depends on DNA methylation and nascent RNA synthesis.

**Fig. 7:**
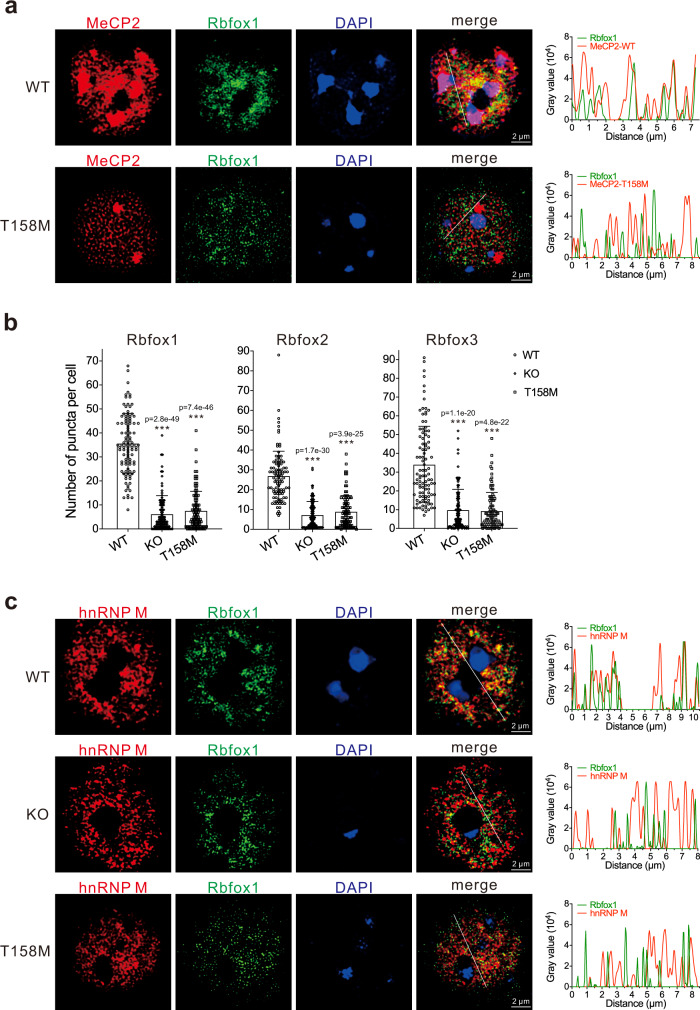
MeCP2 KO and T158M mice show defects in forming nuclear condensates with Rbfox proteins. **a** Representative immunofluorescence microscopic images of endogenous MeCP2 and Rbfox1 in the brains of WT and MeCP2 T158M mice. Scale bar, 2 µm. Line scan graphs are shown on the right. The average Pearson’s *R* values for MeCP2 and Rbfox1 in WT and T158M mice with standard deviations are 0.58 ± 0.12 and −0.31 ± 0.11, respectively (*n* = 30 cells, DAPI-bright regions were excluded during quantitation). **b** The numbers of puncta >0.04 μm^2^ formed by Rbfox1, Rbfox2, and Rbfox3 were counted per cell. Error bars represent standard deviations (*n* = 100 cells; ***p < 0.001, two-sided Student’s *t* test). **c** Representative immunofluorescence microscopic images of endogenous Rbfox1 and hnRNP M in the brains of WT, MeCP2 KO, and T158M mice. Scale bar, 2 µm. Line scan graphs are shown on the right. The average Pearson’s *R* values with standard deviations for hnRNP M and Rbfox1 in WT, MeCP2 KO, and T158M mice are 0.43 ± 0.11, −0.06 ± 0.10, and −0.07 ± 0.10, respectively (*n* = 30 cells). Source data are provided as a Source data file.

Next, we examined whether MeCP2 and Rbfox proteins form condensates in mouse cerebral cortex by performing immunofluorescence staining assays (Supplementary Fig. 15a–c). In WT mice, MeCP2 had strong staining at pericentromeric heterochromatin overlapped with 4,6-diamidino-2-phenylindole (DAPI)-dense regions, as documented earlier^4^. Apart from heterochromatin, the rest of MeCP2 was partially co-localized with Rbfox1/2/3 proteins (Fig. 7a and Supplementary Figs. 15 and 16a, b). A portion of MeCP2 T158M was redistributed to nucleolus as previously reported^35^, and the rest of T158M showed much less co-localization with Rbfox1/2/3 proteins than MeCP2 WT (Fig. 7a and Supplementary Fig. 16a, b). Notably, the numbers of puncta formed by Rbfox proteins >0.04 μm^2^ per cell in WT mice were higher than those in MeCP2 KO or T158M mice (Fig. 7b). We also examined the localization of the LASR component hnRNP M in mouse brains. In WT mice, Rbfox proteins were partially co-localized with hnRNP M, while a clear decrease in their co-localization was observed in MeCP2 KO or T158M mice (Fig. 7c and Supplementary Fig. 17a–c). These data further confirmed the defects in forming the MeCP2/Rbfox/LASR complex in MeCP2 KO and T158M mice.

## Discussion

In this study, we discovered that MeCP2 forms a higher-order assembly with the splicing complex Rbfox/LASR (Figs. 1, 2, and 4 and Supplementary Fig. 6). In cultured human cells and mouse brains, depletion or mutation of MeCP2 led to the dissociation of MeCP2/Rbfox/LASR (Figs. 3a, b and 4b and Supplementary Fig. 6), resulting in decreased binding of Rbfox proteins to their pre-mRNA targets and mis-regulation of alternative splicing (AS; Figs. 3 and 5 and Supplementary Figs. 8 and 9). The scaffold role of MeCP2 in the assembly of Rbfox/LASR correlates with its ability to drive phase condensation. MeCP2 with disease mutations reduced the ability to form condensates both in vitro and in vivo (Figs. 6a, e and 7a and Supplementary Fig. 11e). MeCP2 interacts directly with the multivalent protein RBFOX2 through its MBD and ID (Fig. 2) and forms nuclear condensates with Rbfox proteins in cultured cells and mouse brains (Figs. 6 and 7 and Supplementary Figs. 13 and 16). In MeCP2 KO and T158M mice, Rbfox proteins formed smaller condensates that exhibited less co-localization with LASR component, hnRNP M (Fig. 7b and Supplementary Figs. 16 and 17). Taken together, these data link an impaired function of MeCP2 mutation in splicing control to its defective properties to mediate the higher-order assembly of the MeCP2/Rbfox/LASR complex.

Our data provide an impactful link between Rbfox proteins and RTT. Besides their roles in normal neuronal function, Rbfox proteins have been associated with a range of neurological disorders. Deletion, mutation, and translocation in the *Rbfox1* gene were found in individuals with mental retardation, epilepsy, autism, and development delay^36–38^. Consistently, CNS-specific *Rbfox1* KO displayed seizure and neuronal hyperexcitation phenotype^17^. Deletion in the *Rbfox3* gene was also found in patients with epilepsy^39^. All three Rbfox proteins are underexpressed in individuals with autism spectrum disorder^29,40^. As epilepsy and autistic features are common in RTT, it suggests that defects in the expression/function of Rbfox proteins may contribute to the pathogenesis of RTT and manipulating Rbfox proteins may provide a potential therapeutic strategy for the treatment of RTT. In addition, both Neurexins and Neuroligins are synaptic cell adhesion molecules that connect pre- and post-synaptic neurons at synapses and play critical roles in synaptic formation and *trans*-synaptic signaling. Mutations or alterations in the expression of Neurexins or Neuroligins have been implicated with epilepsy, intellectual disability, and autism spectrum disorders^32^. The functions of AS of exon SS4 in Neurexin genes have been extensively studied and shown to control neuronal synapse specification and memory preservation^41,42^. It will be interesting to further explore the roles of these newly identified splicing targets for Rbfox/LASR in the pathogenesis of RTT.

The assembly of individual biomolecules into a multiunit functional complex at the same time and place is fundamental to nearly all biological processes. Emerging evidence indicates that many membraneless compartments range in size from tens of nm to tens of μm in cells are formed by LLPS, which have been shown to play important roles in a wide variety of cellular processes including autophagy, DNA damage, signal transduction, synaptic development, heterochromatin formation, transcriptional activation, and ribosome biogenesis^43–45^. The number of proteins proved to display liquid-like physical property is growing fast. However, the regulation of the condensate formation and the function of the cellular condensate in gene expression regulation are not well understood. Recently, three studies reported that MeCP2 has the ability to form liquid-like condensate and MeCP2 disease mutations display abolished condensate property^46–48^. Wang et al. showed that MeCP2-mediated condensate formation participates in chromatin compartmentalization by competing with histone H1^46^. Li et al. reported that MeCP2 is a dynamic component of heterochromatin condensates and suggested a link between disruptive condensate properties of RTT mutations and changes in chromatin architecture and transcriptional regulation of RTT^48^. In addition, all three studies found that methylated DNA enhances phase separation of MeCP2 in vitro^46–48^. Here we focus on the nuclear condensates formed by MeCP2, which are not localized in the DAPI-bright heterochromatin but localized in the DAPI-weak euchromatin. Both MeCP2 and Rbfoxs are multivalent proteins and carry IDR or LCR. We show that MeCP2 is able to co-phase separate with Rbfox proteins in vitro and forms condensates with Rbfox proteins in both cultured human cells and mouse brains. Results from 5-Aza and DRB treatment (Supplementary Figs. 5 and 14) further suggest that these nuclear condensates are formed co-transcriptionally and primed by DNA methylation or MeCP2 binding to chromatin. Importantly, loss of or disruption of condensate properties by MeCP2 KO or disease-causing mutations displayed defects in forming MeCP2 condensates with these splicing regulators. Together, these findings link RTT to an impaired function of MeCP2 in splicing control through its role in nucleating Rbfox/LASR macromolecule assembly.

In metazoan, the majority of splicing events occur co-transcriptionally^49^. Our data provide evidence that MeCP2 functions in co-transcriptional regulation of AS by concentrating local splicing regulators on the chromatin via condensate formation. Previous studies on co-transcriptional regulation of splicing by MeCP2 show that it binds to methylated DNA and affects the elongation rate of PoI ΙΙ to modulate exon inclusion or intron retention^50,51^. Here, our results suggest that MeCP2 acts as a hub for nucleating a group of RNA-binding proteins (RBPs) with a high local concentration, while loss of or mutation of MeCP2 has defects in promoting higher-order assemblies, leading to the reduced binding of splicing regulators to their targets or the remodeling of local interaction networks between splicing regulators and their bound RNAs. Recently, multivalent assemblies of RBPs into higher-order protein complexes have been shown to expand the regulatory capacity of global splicing control^27,52^. Considering that MeCP2 is a multivalent protein and its broad binding to chromatin, further research is warranted to investigate whether MeCP2 recruits any other chromatin remodeling factors or post-transcriptional regulators via forming the higher-order assemblies on chromatin and whether these condensates mediate the molecular functions of MeCP2 underlying the pathogenesis of RTT.

## Methods

### Mice

Female *Mecp2*^*fl/fl*^ mice bearing loxP sites flanking exon 3 of the *Mecp2* gene (B6.129S4-*Mecp2*^*tm1Jae*^/Mmucd)^7^ from MMRRC and male *NesCre8* mice were crossed to produce neuronal-specific *Mecp2* KO mice. MeCP2-GFP-T158M^9^ mice (B6.129P2(Cg)-*Mecp2*^*tm4.1Bird*^/J) were purchased from the Jackson Laboratory (#026762). All animals were housed in a pathogen-free facility at an ambient temperature ~22 °C with a humidity of 50% and a 12-h light/12-h dark cycle. Brain tissues were obtained from 6-week-old male mice in this study. All mouse experiments were approved by the Institutional Animal Care and Use Committee (IACUC) at the Shanghai Institute of Biochemistry and Cell Biology, Chinese Academy of Sciences and conducted in accordance with the guidelines of IACUC.

### Plasmid construction

To generate the plasmid for eukaryotic expression of FLAG-MeCP2, PCR fragments encoding FL MeCP2 and its mutation derivatives amplified from HKE293T cell cDNA were inserted into p3×FLAG-CMV-10 (Sigma) between *Eco*RI and *Xba*I sites. To construct expression plasmid for RBFOX2-FLAG, PCR fragments encoding FL RBFOX2 amplified from HEK293T cell cDNA were inserted into pcDNA3 (Invitrogen) between *Hin*dIII and *Xho*I sites.

To construct plasmids for protein purification from bacteria, PCR fragments encoding WT, R106W, T158M, ∆MBD, ∆ID, or ∆TRD MeCP2 along with EGFP-twin-strep were inserted into pGEX-6P-1 (Pharmacia), PCR fragments encoding Rbfox2 or hnRNPA1 along with mCherry were inserted into pET28a (Novagen) between *Bam*HI and *Xho*I sites, PCR fragments encoding MBD, ID, or MBD + ID were inserted into pGEX-5X-2 (Cytiva) between *Bam*HI and *Eco*RI sites, and PCR fragment encoding RBFOX2 was inserted into pET28a between *Bam*HI and *Xho*I sites.

The splicing minigene constructs contain the alternative exon together with its flanking constitutive exons and introns. For *Nrxn3* WT minigene, intron sequences were shortened from 250 nt downstream of the 5’ splicing site (5’SS) to 350 nt upstream of the 3’SS. For *Nlgn1* WT minigene, intron sequences were deleted from 250 nt downstream of the 5’SS to 250 nt upstream of the 3’SS. In *Nrxn3* and *Nlgn1* mutant constructs, RBFOX2-binding sites downstream of the alternative exon were mutated. PCR fragments containing mouse *Nrxn3* and *Nlgn1* sequences generated by PCR mutagenesis method were inserted into pcDNA3 between *Hin*dIII and *Xho*I sites. The sequences of all the oligonucleotides used in this study are listed in Supplementary Table 1.

### Western blotting

Protein samples were separated by sodium dodecyl sulfate (SDS)–polyacrylamide gel electrophoresis (PAGE) followed by gel transfer to nitrocellulose membrane (Cytiva). The membranes were sequentially incubated with primary antibodies and horseradish peroxidase (HRP)-conjugated secondary antibodies followed by detection with enhanced chemiluminescence system (Millipore) using Amersham Imager 680 (Cytiva). The primary antibodies used in this study were anti-FLAG (Sigma-Aldrich, F3165, 1:8000 diluted), anti-GAPDH (ImB, MM001, 1:3000 diluted), anti-MeCP2 (Diagenode, pAb-052-050), anti-Rbfox1(Millipore, MABE159), anti-Rbfox2 (Bethyl, A300–864A), anti-Rbfox3 (Millipore, MAB377), anti-Matrin3 (Bethyl, A300–591A), anti-hnRNP M (Santa Cruz, sc-20001), anti-hnRNP A1 (Santa Cruz, sc-32301), anti-hnRNP C (Abclonal, A0057), anti-hnRNP H1 (Abclonal, A5924), anti-hnRNP U (Abclonal, A9907), anti-hnRNP F (Abclonal, A5505), anti-NF110 (Abclonal, A2496), anti-hnRNP K (Abclonal, A1701), anti-histone H3 (Abcam, ab1791), anti-DDX5 (Abcam, ab21696), and anti-GFP (Proteintech, 66002-1-Ig). Dilutions of all primary antibodies were 1:1000 if not specifically mentioned. HRP-conjugated secondary antibodies (1:2500 diluted) were anti-mouse IgG (Promega, W4021), anti-rabbit IgG (Promega, W4011), and conformation-specific anti-rabbit IgG (Cell Signaling, 3678).

### Preparation of HMW extract

Cultured cell pellets resuspended in ten volumes of cold hypotonic buffer (10 mM HEPES-KOH pH 7.5, 1.5 mM MgCl_2_, 10 mM NaCl, 1.25× cOmplete^TM^ protease inhibitors (Roche), 0.5 mM dithiothreitol (DTT), 0.15% NP-40) or mouse tissues homogenized in cold brain lysis buffer (10 mM HEPES-KOH pH 7.5, 5 mM CaCl_2_, 3 mM MgCl_2_, 0.1% NP-40, 0.1 mM EDTA, 0.32 M Sucrose, 1 mM DTT) by pushing through 26 G needles were centrifuged at 956 × *g* for 8 min at 4 °C followed by incubation with ten volumes of HMW lysis buffer (20 mM HEPES-KOH pH 7.5, 150 mM NaCl, 1.5 mM MgCl_2_, 0.5 mM DTT, 1.25× cOmplete^TM^ protease inhibitors, 0.6% Triton X-100) for 5 min. Soluble fractions and pellets were separated by centrifugation at 20,000 × *g* for 5 min at 4 °C. The pellets were lysed with an equal volume of HMW lysis buffer containing 5 U/µl of Benzonase (Sigma, E1014-25KU) at 25 °C on a rotator for 20 min, and the HMW extracts were cleared by centrifugation at 20,000 × *g* for 10 min at 4 °C.

### Immunoprecipitation

HMW extracts isolated from cultured cells or mouse tissues were incubated at 4 °C for 3 h with FLAG M2 Agarose beads (Sigma, A2220) or the appropriate antibodies previously immobilized on Protein G Agarose Beads (Roche, 11243233001). The beads were subsequently washed three times with wash buffer (50 mM Tris·Cl pH 7.4, 300 mM NaCl, 0.1% NP-40, 1 mM DTT, 1 mM phenylmethanesulfonylfluoride (PMSF)). Proteins were separated by SDS-PAGE followed by western blotting.

### Glycerol gradients

HMW extracts were loaded and resolved on 10–50% glycerol gradients containing 20 mM HEPES-KOH pH 7.5, 150 mM NaCl, 0.5 mM DTT, 1.25× cOmplete^TM^ protease inhibitors in 11 × 34 mm centrifuge tubes (Beckman Coulter). The gradients were then spun in an S55S rotor (Hitachi) at 130,000 × *g* for 15 h at 4 °C.

### GST pull-down

GST or GST-tagged proteins immobilized on 10 μl of glutathione-Sepharose 4B (Cytiva, 17-0756-01) were incubated with His-Rbfox2 for 3 h at 4 °C in N150 buffer (50 mM Tris·Cl pH 7.4, 150 mM NaCl, 0.1% NP-40, 1 mM DTT) followed by washing four times with 1 ml N150 buffer. The bound proteins were fractionated by 10% SDS-PAGE and visualized by Coomassie blue staining.

### Affinity pull-down by biotinylated double-stranded DNA (dsDNA) oligonucleotides

Single-stranded biotinylated DNA oligonucleotides (sequences in Supplementary Table 1) were annealed into dsDNA in PCR buffer (50 mM KCl, 10 mM Tris·Cl pH 8.3, 1.5 mM MgCl_2_). In all, 1 μg annealed dsDNA was immobilized on 10 μl Dynabeads^TM^ M-280 Streptavidin (Invitrogen, 11206D) in BW Buffer (10 mM Tris·Cl pH 7.4, 1 mM EDTA, and 1 M NaCl) at room temperature for 15 min. After washing with BW Buffer, beads were incubated with 5 μg GST or GST-MBD-ID proteins with or without His-Rbfox2 for 3 h at 4 °C in N150 buffer followed by washing with N150 buffer. The bound proteins were fractionated by 10% SDS-PAGE and visualized by Coomassie blue staining.

### Generation of HEK293T KO or KI cell lines by CRISPR/Cas9

To generate HEK293T MeCP2 KO cell lines, CRISPR guide RNA (gRNA) sequences were designed by http://crispr.mit.edu. Oligonucleotides carrying two pairs of gRNA sequence were annealed and cloned into px330-mCherry vector (Addgene), respectively. HEK293T cells were transfected with sgRNA expression plasmids. Forty-eight hours post-transfection, mCherry-positive cells were sorted into 96-well plates using FACSAria SORP (BD Biosciences). Single colonies were validated by genotyping, Sanger sequencing, and western blot analysis.

To generate RBFOX2 ΔCTD KI HEK293T cell lines, CRISPR guide sequences targeting the flanking regions of exon 10 and exon 14 of *RBFOX2* were designed to delete RBFOX2 CTD encoding sequence. To construct the donor plasmid for KI, the left homology and right homology arms containing ~1500 bp sequences flanking intron 9 and exon 14, respectively, were inserted into pGEM^®^-T Easy vector (Promega, PR-A1360). HEK293T cells were transfected with two sgRNA CRISPR vectors together with the donor plasmid at a ratio of 1:1:2. Positive colonies were sorted and validated as described above. The resulting KI cells express a truncated RBFOX2 protein (the first 326 aa of RBFOX2) with an SV40 NLS introduced upstream of the stop codon.

To generate MeCP2-eGFP with/without RBFOX2-mCherry KI HEK293T cell lines, donor plasmids containing the corresponding left and right homology arms as well as the coding sequence of eGFP or mCherry were generated. HEK293T cells were transfected with MeCP2-eGFP sgRNA CRISPR vector and donor plasmid at a ratio of 2:1. eGFP-positive colonies were sorted into 96-well plates and further validated by fluorescence microscope observation and Sanger sequencing. Validated MeCP2-eGFP-KI cell lines were further transfected with RBFOX2-mCherry sgRNA CRISPR vector and its corresponding donor plasmid at a ratio of 2:1. eGFP and mCherry double positive colonies were sorted and sequencing validated.

### RNA-seq and data analysis

Total RNAs were isolated from cultured cells or cerebral cortex tissues from MeCP2 mutant mice and their WT littermates at age 6 weeks using TRIzol^TM^ Reagent (Invitrogen, 15596026) and processed for paired-end (2 × 150 nt) RNA-seq on an Illumina X Ten platform according to the manufacturer’s instruction. The paired-end raw reads were first cleaned by Trimimomatic^53^. After trimming the adapter sequence, removing low-quality bases, and filtering short reads, cleaned reads pairs were retained for further analysis. The genome sequence and gene annotation of human (GRCh38) and mouse (GRCm38) were downloaded from GENCODE^54^. Cleaned reads were mapped to the genome sequence by HISAT^55^ with default parameters. Numbers of reads that were mapped to each gene were calculated with the htseq-count script in HTSeq^56^. AS events in RNA-seq data were detected by rMATS^57^ and PAIRADISE^58^ with junction reads coverage >15. The PSI (percent spliced in) difference of a given AS event between two conditions was considered to be significant if the false discovery rate calculated by rMATS was <0.05 and the ΔPSI was >10.

### iCLIP-seq and data analysis

iCLIP-seq was performed as previously described^59^ with several modifications. Briefly, HEK293T WT or MeCP2 KO cells were irradiated once with 125 mJ/cm^2^ at 254 nm. The iCLIP lysis buffer (50 mM Tris·Cl pH 7.4, 100 mM NaCl, 1% NP-40, 0.1% SDS, 0.5% sodium deoxycholate, 1× cOmplete^TM^ protease inhibitors) was added to resuspend cell pellets. Cell lysates were sonicated in a Bioruptor (Diagenode), and then RNAs were partially fragmented with either high or low concentration of RNase A (Qiagen, 19101). The RNA–protein complexes were immunoprecipitated with anti-RBFOX2 antibody immobilized on protein G dynabeads (Invitrogen, 10004D), and washed with High salt wash buffer (50 mM Tris·Cl pH 7.4, 1 M NaCl, 1 mM EDTA, 1% NP-40, 0.1% SDS, 0.5% Sodium Deoxycholate) and PNK buffer (20 mM Tris·Cl pH 7.4, 10 mM MgCl_2_, 0.2% Tween-20). 3’ End of RNAs were dephosphorylated and ligated to a L3-linker. RNAs were then radioactively labeled by T4 polynucleotide kinase (Thermo, EK0031), and transferred from the NuPAGE^TM^ 4–12% Bis-Tris protein gels (Invitrogen) to a Protran BA 85 nitrocellulose membrane (Whatman). The RNA–protein complexes from a low concentration of RNase A treatment were recovered from the membrane and reverse transcribed with RTclip2.0 primer. cDNA was purified and ligated at 5’ end with Lclip2.0 linker containing a certain barcode. For iCLIP cDNA library amplification, cDNA was first amplified with P5Solexa_s and P3Solexa_s, and PCR products were size selected with ProNex^®^ chemistry. Products from the first amplification were then re-amplified with P5Solexa and P3Solexa and size selected. The cDNA library was processed on an Illumina X Ten sequencer.

The iCLIP data were processed from raw reads to RBP-binding sites by the pipeline as previously described^60^. Briefly, the barcoded regions in the reads were filtered by FASTX-Toolkit. The demultiplexing and adapter trimming were performed by Flexbar^61^. Only trimmed reads with a length at least 15 nt were kept for further analysis. The individual FASTQ files for each sample were mapped to the human reference genome (GRCh38) by STAR^62^. The technical duplicates were removed using UMI-tools^63^. After deduplication, the crosslink events were transformed from the mapped reads by extracting the position upstream of the 5’ end of the reads with BEDtools^64^. The crosslink sites with significantly enriched crosslink events were identified by PureCLIP^65^ and post-processed by the steps as previously described^60^.

### RNA map

The crosslink events located within one annotated gene were normalized by the total count of the respective gene and the gene length^66^. The total number of normalized crosslink events relative to the 3’SS and 5’SS of the enhanced and silenced skipped exon (SE) events were shown in the RNA map. We used a 30 nt sliding window with 1 nt step to detect the regions in which crosslink events were significantly different between WT and KO samples. In each step, we performed *t* test with the number of crosslink events of two samples and calculated the fold change. The windows with Bonferroni adjusted *p* value < 0.01 and fold change >2 were merged and shown as a red box in the RNA map (Fig. 3f).

### RNA immunoprecipitation

The freshly dissected cerebral cortex was partially triturated in ice-cold dissection buffer (161 mM NaCl, 5 mM KCl, 1 mM MgSO_4_, 3.7 mM CaCl_2_, 5 mM HEPES, 5.5 mM glucose) and transferred to a 10-cm cell culture plate followed by 3 pulses of UV irradiation at 160 mJ/cm^2^ and homogenizing in iCLIP lysis buffer. Homogenized samples were sonicated with Bioruptor (Diagenode), and then RNAs were partially fragmented with RNase A. After centrifugation at 20,000 × *g* for 20 min, an aliquot (10%) of supernatant was saved as input sample. The remaining supernatant was immunoprecipitated with Rabbit IgG (ImB, IL003), anti-Rbfox1, anti-Rbfox2, anti-Rbfox3, or anti-hnRNP M (Abclonal) antibodies immobilized on protein G dynabeads. Beads bound with RNA–protein complexes were washed with High salt wash buffer and PNK buffer, respectively. Immunoprecipitated RNAs were isolated using TRIzol^TM^ Reagent. and then reverse-transcribed into the first-strand cDNAs using Random primer and Superscript III reverse transcriptase (Invitrogen, 18080093). The cDNAs were further analyzed by real-time qPCR using iTaq™ Universal SYBR Green® Supermix (Bio-Rad, 1725121) on a LightCycler® 96 Instrument (Roche, 05815916001).

### Chromatin immunoprecipitation

The freshly dissected cerebral cortex was partially triturated in phosphate-buffered saline (PBS) and crosslinked with 1% formaldehyde at room temperature for 20 min. Crosslinking was then quenched with 0.125 M glycine for 10 min at room temperature. Crosslinked tissue was then homogenized in Nuclei lysis Buffer (50 mM Tris·Cl pH8.0, 10 mM EDTA, 1% SDS, and 1× cOmplete^TM^ protease inhibitors). Soluble chromatin was prepared by sonication and the final average size of DNA fragment was 150–300 bp. After centrifugation at 20,000 × *g* for 20 min, an aliquot (5%) of supernatant was saved as input sample. The remaining supernatant was pre-cleared and diluted with 2× ChIP dilution buffer (0.02% SDS, 2.2% Triton X-100, 2.4 mM EDTA, 33.4 mM Tris·Cl pH 8.0, 334 mM NaCl). Diluted chromatin was immunoprecipitated with Rabbit IgG, anti-Rbfox1, anti-Rbfox2, anti-Rbfox3, anti-hnRNP M, or anti-MeCP2 antibodies immobilized on pre-blocked protein G dynabeads. Beads bound with chromatin–protein complexes were washed three times with TSE I buffer (20 mM Tris·Cl pH 8.0, 150 mM NaCl, 2 mM EDTA, 1% Triton X-100, 0.1% SDS), three times with TSE II buffer (20 mM Tris·Cl pH 8.0, 500 mM NaCl, 2 mM EDTA, 1% Triton X-100, 0.1% SDS), once with Wash buffer III (10 mM Tris·Cl pH 8.0, 250 mM LiCl, 1 mM EDTA, 1% NP-40, 1% sodium deoxycholate) and once with TE buffer. The immunoprecipitated sample was eluted from beads with Elution buffer (10 mM Tris·Cl pH 8.0, 1 mM EDTA, 1% SDS). After decrosslinking, RNase A digestion, and proteinase K treatment, recovered DNA was further analyzed by qPCR.

### ChIP-seq/WGBS and data analysis

HEK293T cells were crosslinked with 1% formaldehyde at room temperature for 15 min and then quenched with 0.125 M glycine for 10 min at room temperature. Crosslinked cells were then homogenized in cell lysis buffer (10 mM Tris·Cl pH8.0, 10 mM NaCl, 1.5 mM MgCl_2_, 0.1% Triton X-100). After centrifugation at 956 × *g* for 10 min, pellets were further homogenized in nuclei lysis buffer. Sonicated chromatin with an average size of 150–300 bp was cleared by centrifugation, an aliquot (5%) of supernatant was saved as input sample. Diluted chromatin was immunoprecipitated with MeCP2 antibody immobilized on pre-blocked protein G dynabeads. Beads bound with chromatin–protein complexes were washed three times with TSE I buffer, three times with TSE II buffer, once with Wash buffer III and once with TE buffer. The immunoprecipitated sample was eluted from beads with Elution buffer. After decrosslinking, RNase A digestion, and proteinase K treatment, recovered Input and ChIPed DNA were then processed for paired-end (2 × 150 nt) RNA-seq on an Illumina X Ten platform according to the manufacturer’s instruction of NEBNext Ultra II DNA Library Prep Kit for Illumina (NEB, E7645). The paired-end ChIP-Seq reads were mapped to the human reference genome (GRChg38) by Bowtie2^67^ with default parameters. The aligned reads with low mapping quality (MAPQ < 30) or multiple positions on the genome were removed by SAMtools^68^. Enriched peaks were identified by MACS^69^ with default parameters. The genomic positions of the peaks were annotated by annotatePeak function in ChIPseeker package^70^. Read coverages along the genome were calculated and normalized to reads per million (RPM) by BEDtools^64^. The WGBS data of HEK293 are from GEO database (GSE94055). We randomly chose the background splicing events that were located on the same genes of the regulated events, then calculated the average normalized MeCP2-binding intensities (RPM) and CpG methylation levels along the flanking 150 nt regions of the SEs for the regulated and random events, respectively. We also calculated the 99% confidence interval of the random events for ChIP-seq and CpG methylation data, respectively. The confidence interval was calculated by CI function from Rmisc package in R.

### In vivo splicing and RT-PCR

Minigene plasmids were co-transfected with either empty pcDNA3 vector or pcDNA3-RBFOX2-FLAG expression plasmid into HEK293T cells. Total RNAs were isolated with TRIzol^TM^ Reagent 40 h after transfection and subjected to RT-PCR analysis using BGH as RT primer and gene-specific primers for PCR. Exon inclusion ratios were quantified using GelAnalyzer.

### Protein expression and purification

Recombinant MeCP2 and RBFOX2 proteins were expressed in *Escherichia* *coli* strain BL21 (DE3) Gold. Cultures were grown in LB media at 37 °C to OD_600_ of 0.6, then induced by 0.2 mM IPTG at 18 °C overnight. The cells were harvested by centrifugation at 4 °C and disrupted by French Press (JNBio) in buffer 1 (20 mM Tris·Cl pH 8.0, 500 mM Imidazole, 1 mM PMSF) for GST-MeCP2 FL/∆MBD/∆ID/∆TRD/R106W/T158M-eGFP-twin-strep proteins and His-RBFOX2-mCherry protein. After centrifugation at 16,000 × *g* for 1 h, the supernatant of recombinant MeCP2 proteins was loaded onto an HisTrap HP column (Cytiva, 17524801). After extensive washing with buffer 1, the target protein was eluted by buffer with a gradient of imidazole from 25 to 500 mM. GST-tag was cleaved at 4 °C by 3 C protease. MeCP2 proteins were further loaded onto a Strep-Tactin^®^XT column (IBA, 2-4022-001). After extensive washing with buffer W (100 mM Tris·Cl pH 8.0, 150 mM Imidazole, 1 mM EDTA), MeCP2 proteins were eluted by buffer W supplemented with 50 mM biotin, and then the eluate was further flowed through a HiTrap Q HP anion exchange chromatography column (Cytiva, 17115401) to remove impure proteins and loaded onto a HiTrap SP HP chromatography column (Cytiva, 17115201). The target protein was eluted by buffer with a gradient of NaCl from 100 mM to 1 M. The MeCP2 proteins were dialyzed to the final buffer containing 300 mM NaCl and 20 mM Tris·Cl pH 8.0. The supernatant of recombinant RBFOX2 protein was loaded onto a His-Trap HP column, and the eluate was further loaded onto a HiTrap Heparin HP affinity column (Cytiva, 17040703). His-RBFOX2-mCherry protein was dialyzed to the final buffer containing 150 mM NaCl and 20 mM Tris·Cl pH 8.0.

Recombinant GST-MBD/ID/MBD-ID proteins were expressed in the *E. coli* strain BL21(DE3) Gold. The cells were harvested by centrifugation at 4 °C and disrupted by French Press in buffer S (20 mM Tris·Cl pH 8.0, 500 mM NaCl, 1 mM PMSF). After centrifugation at 16,000 × *g* for 1 h, the supernatant was loaded onto a GSTrap HP column (Cytiva, 17528101). After extensive washing with buffer S, the target protein was eluted using buffer S supplemented with 10 mM L-Glutathione reduced (GSH). The target proteins were loaded on a HiTrap SP HP column in Buffer A (100 mM NaCl, 20 mM Tris·Cl pH 8.0, 0.1% 2-mercaptoethanol) and were gradient eluted with Buffer B (1 M NaCl, 20 mM Tris·Cl pH 8.0, 0.1% 2-mercaptoethanol). The target proteins were further purified by passage through a HiLoad 16/60 Superdex G75 column (Cytiva, 28989333) with buffer GF (10 mM Tris·Cl pH 8.0, 500 mM NaCl, 1 mM DTT). The fractions containing the target proteins were pooled and concentrated for further use.

### Droplet assembly

Recombinant eGFP or mCherry fusion proteins were diluted from a high salt storage buffer to different concentrations in a buffer containing 20 mM Tris·Cl pH 7.5, 0.1 mM PMSF, and indicated salt concentration. For 150 mM salt condition, crowding agent PEG-8000 was added with a final concentration of 10%. The protein solution was immediately loaded onto a homemade chamber comprising a glass slide with a coverslip attached to two parallel strips of double-sided tape. Slides were imaged within 10 min under a Zeiss LSM 880 with a ×63 immersion objective.

### FRAP assay

In vivo and in vitro FRAP experiments were performed on a White Light Laser Confocal microscope (Leica TCS SP8 X) with a ×63 (in vivo) or ×100 (in vitro) immersion objectives and operated with the LAS-X imaging software. For in vitro FRAP assay, the region of interest (ROI) was photobleached with a 488-nm laser (2 repeats, 100% intensity, dwell time 1 s) and images were collected every 5 s. For in vivo FRAP, cells were seeded on 29 mm no.1.5 glass-bottom dishes (Cellvis). The culture medium was replaced by phenol red-free Dulbecco’s modified Eagle’s medium (DMEM; GIBCO) supplemented with 10% fetal bovine serum (FBS) before imaging. Images were acquired on a White Light Laser Confocal microscope Leica TCS SP8 X under FRAP mode. The ROI was photobleached with a 488-nm laser using 75% laser power (dwell time 1 s), and images were collected every 5 s. Fluorescence intensity within the ROI was obtained for each experiment. Background intensity was subtracted, and fluorescence intensity values were normalized relative to pre-bleaching time points.

### Immunofluorescence

Six-week-old mice were transcardially perfused with 4% paraformaldehyde (PFA) in PBS, and the brains were dissected and post-fixed in PBS containing 4% PFA overnight at 4 °C. Fixed tissues were dehydrated with 30% sucrose and embedded for cryostat sectioning. Embedded tissues were sectioned coronally at a thickness of 40 µm. Free-floating brain sections were permeabilized/blocked in PBS containing 0.75% Triton X-100 and 5% bovine serum albumin (BSA) for 1.5 h at room temperature. Pretreated sections were incubated in diluted primary antibodies (MeCP2 1:500, Rbfox1 1:100, Rbfox2 1:100, Rbfox3 1:100, hnRNP M 1:500) in PBS containing 1% BSA overnight at 4 °C and then incubated in diluted fluorescent secondary antibodies (1:500) and DAPI (Invitrogen, D1306) for 1 h at room temperature. Stained sections were finally mounted onto glass slides with ProLong^TM^ Diamond Antifade Mountant (Invitrogen, P36965). Immunofluorescence of HEK293T cells was performed as described above with several modifications. Briefly, cells seeded on the coverslips were fixed with 4% PFA in PBS for 10 min at room temperature, permeabilized using 0.5% Triton X-100 in PBS for 5 min, and then blocked with PBS containing 10% BSA for 1 h at room temperature. Primary antibodies were diluted (MeCP2 1:500, Rbfox2 1:100) with PBS containing 1% BSA and incubated for 1.5 h at room temperature. Fluorescent secondary antibodies were 1:500 diluted in PBS containing 1% BSA and incubated for 1 h at room temperature. The nuclei were stained with DAPI for 5 min and finally mounted onto glass slides. Fluorescent secondary antibodies used in immunofluorescence were goat anti-rabbit secondary antibody, Alexa Fluor 488 (Thermo, A-11008), goat anti-mouse secondary antibody, Alexa Fluor 488 (Thermo, A-11001), goat anti-mouse secondary antibody, Cyanine3 (Thermo, A10521), and goat anti-rabbit secondary antibody, Alexa Fluor 647 (Thermo, A-21244). Two or three brain slices per mouse (*n* = 3 mice per strain) were prepared as described above. Low magnified images of cortical layers were acquired on a White Light Laser Confocal microscope Leica TCS SP8 X with a ×10 objective under Mark & Find and Tile Scan mode. High-resolution images were acquired on a White Light Laser Confocal microscope Leica TCS SP8 X with a ×63 objective under HyVolution mode and then were deconvolved using Huygens Essential Automatic approach provided by the LAS-X imaging software. Raw images were then post-processed using Fiji Is Just ImageJ.

### Drug treatments

For 5-Aza treatment, cells were treated with 5 μM 5-Aza or the same volume of dimethyl sulfoxide (DMSO) for 72 h. Fresh medium containing 5-Aza or DMSO were replaced every 24 h. For DRB treatment, cells were treated with 100 μM DRB or the same volume of DMSO for 2 h. For DRB washout experiment, cells were treated with DRB for 2 h as described above and then washed with fresh medium and left to recover in fresh medium for 2 h.

### Live imaging

Cells were seeded on 29 mm no.1.5 glass-bottom dishes (Cellvis). The culture medium was replaced by phenol red-free DMEM supplemented with 10% FBS before imaging. Images were acquired on the White Light Laser Confocal microscope Leica TCS SP8 X with a ×63 objective and operated with the LAS-X imaging software. Raw images were post-processed using Fiji Is Just ImageJ.

### Immunofluorescence quantification analysis

Fiji’s plugin Coloc2 was applied in the analysis of co-localization. Individual cells were selected as a ROI, and Pearson’s correlation coefficient was quantified. For quantification of nuclear puncta number, automatic thresholds were calculated for individual deconvolved images. Numbers of nuclear puncta in each cell were automatically counted using Fiji’s Analyze Particles tool, and only particles with a size >0.04 µm^2^ was counted as a nuclear punctum.

### Statistics and reproducibility

Statistical parameters were defined and reported either in individual figures or corresponding figure legends. Statistical analyses were performed using the two-sided Student’s *t* test in Prism 8.0 (Graphpad) or Excel (Microsoft).

### Reporting summary

Further information on research design is available in the Nature Research Reporting Summary linked to this article.

## Supplementary information

Supplementary Figures and Table

Dataset 1

Dataset 2

Dataset 3

Dataset 4

Dataset 5

Description of additional supplementary files

Reporting Summary

## Data Availability

Sequencing data generated in this study are available in the Gene Expression Omnibus under accession number GSE142716. The mass spectrometry data are available at iProX database with the dataset identifier PXD021650. The data supporting the findings of this study are available from the corresponding authors upon reasonable request. Source data are provided with this paper.

## Source data

Source Data

## Code availability

R code to perform RNA map analysis is available at https://github.com/HJY-Lab/MeCP2_splicing (10.5281/zenodo.5336258).
