# Supplementary material for: Rett syndrome linked to defects in forming the MeCP2/Rbfox/LASR complex in mouse models
Source: Nat Commun. 2021 Oct 1;12:5767. doi: 10.1038/s41467-021-26084-3 (PMC8486766; doi:10.1038/s41467-021-26084-3)
Supplement: Supplementary file 1 — Supplementary Figures and Table [file 41467_2021_26084_MOESM1_ESM.pdf]

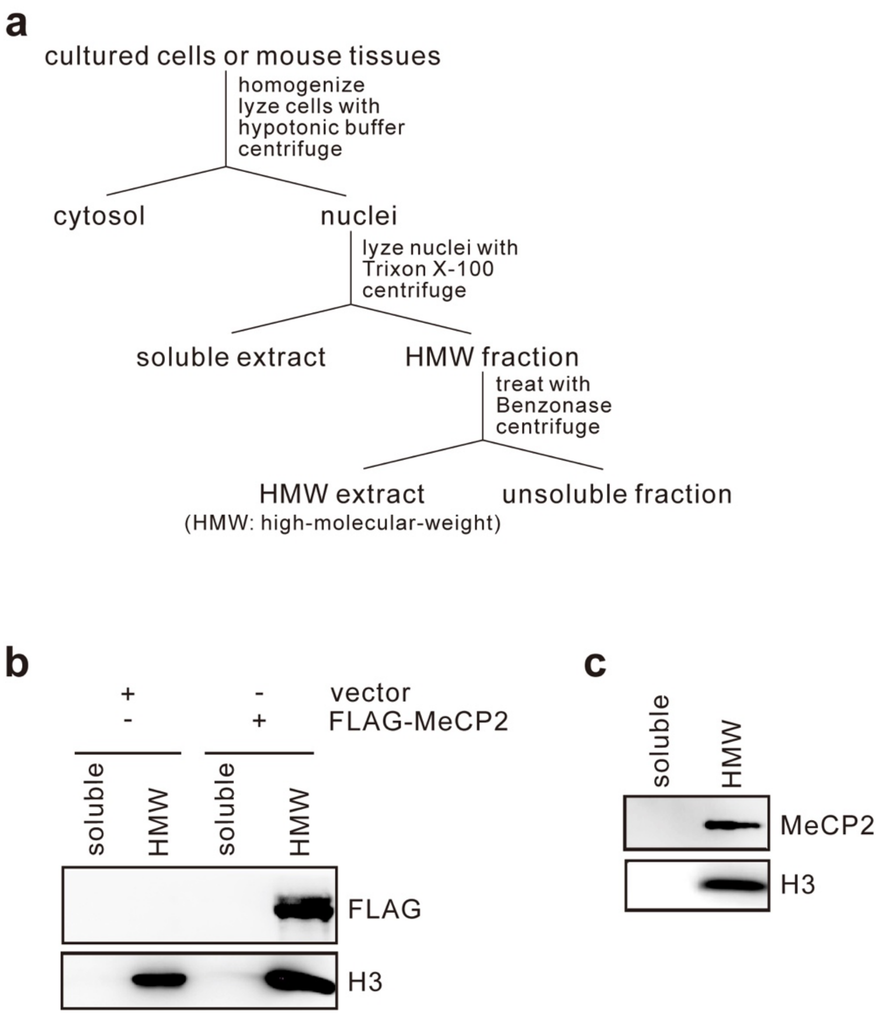

Supplementary Fig. 1 Preparation of HMW extracts from HEK293T cells and mouse brain tissue. a Flow chart depicting the preparation of the soluble and HMW extracts. **b**, **c** Western blot analyses of MeCP2 and H3 in the soluble and HMW extracts from HEK293T cells expressing FLAG-tagged MeCP2 (**b**) or from mouse cerebral cortex (**c**). Source data are provided as a Source Data file.

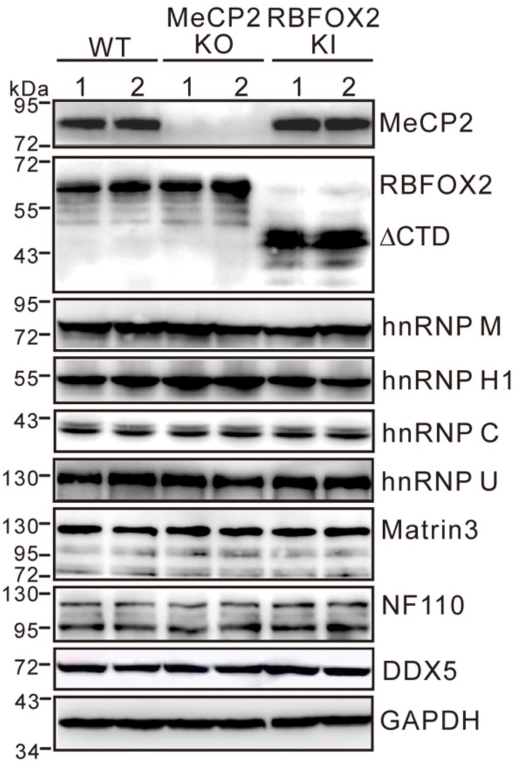

Supplementary Fig. 2 Establishment of MeCP2 KO and RBFOX2 ΔCTD KI HEK293T cells. Western blot analyses of MeCP2, RBFOX2, and LASR components in WT, MeCP2 KO and RBFOX2 KI HEK293T cells. Source data are provided as a Source Data file.

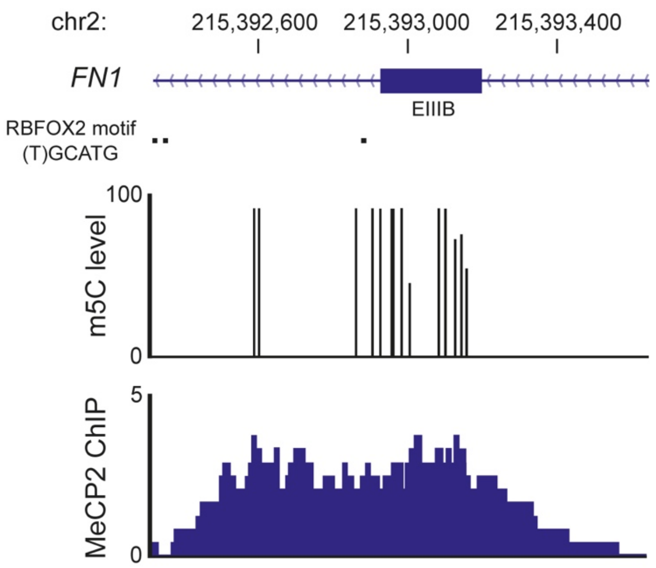

Supplementary Fig. 3 The m5C level and MeCP2 binding profiles at the locus of a splicing target of the MeCP2/Rbfox/LASR. The WGBS and MeCP2 ChIP-seq tracks for *FN1* exon EIIIB and its flanking introns (chr2: 215392931-215393203, minus strand, hg38).

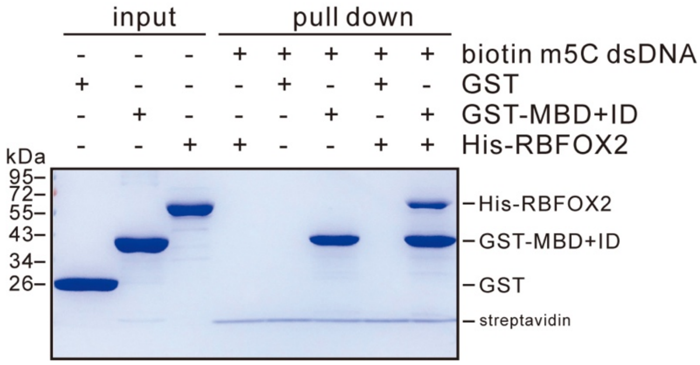

Supplementary Fig. 4 MeCP2 truncated mutant (MBD+ID) interacts both methylated DNA and RBFOX2. Biotinylated dsDNA oligonucleotides containing m5C immobilized on streptavidin beads pulled-down His-tagged RBFOX2 in the presence of GST-tagged MBD+ID but not of GST protein. Source data are provided as a Source Data file.

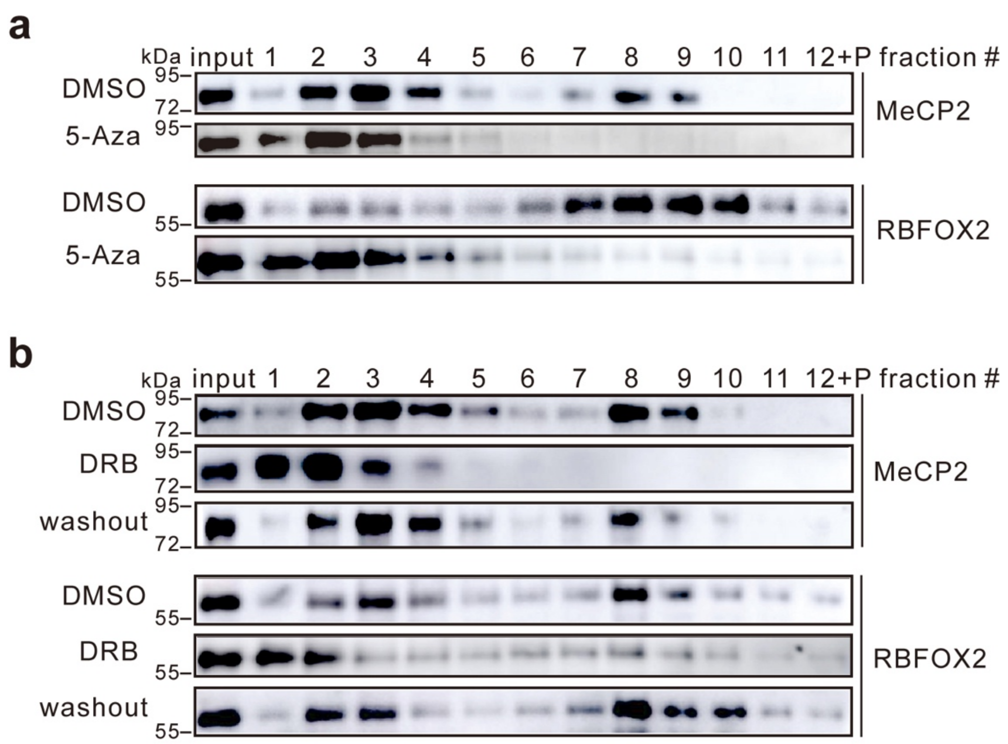

Supplementary Fig. 5 The formation of MeCP2/Rbfox/LASR complex depends on DNA methylation and nascent RNA synthesis. a Sedimentation profiles of MeCP2 and RBFOX2 in the HMW extract from HEK293T cells treated with DMSO or 5-Aza on 10-50% glycerol gradients. **b** Sedimentation profiles of MeCP2 and RBFOX2 in the HMW extract from HEK293T cells treated with DMSO, DRB, or DRB followed by washout on 10-50% glycerol gradients. Source data are provided as a Source Data file.

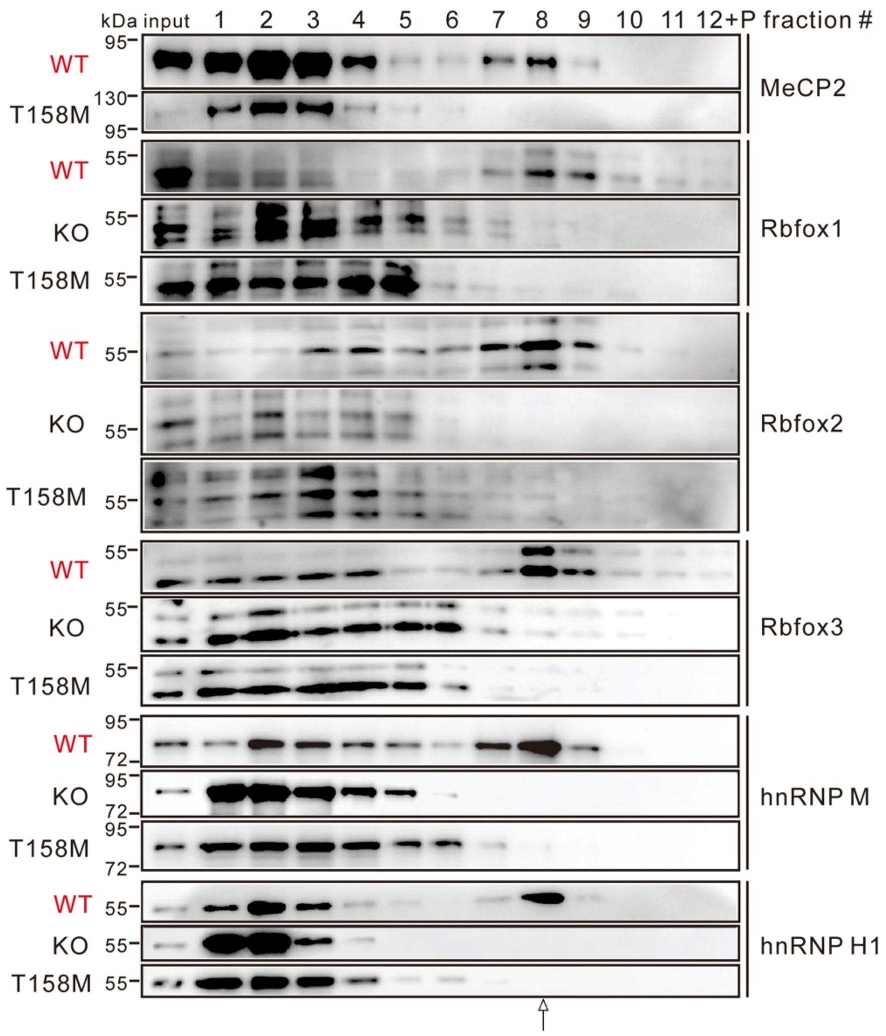

Supplementary Fig. 6 Disruption of Rbfox/LASR complex in the cerebellum of MeCP2 KO and T158M mice. Sedimentation profiles of MeCP2, Rbfox proteins, and LASR components in the HMW extract from the cerebellum of WT, MeCP2 KO and T158M mice on 10-50% glycerol gradients. Source data are provided as a Source Data file.

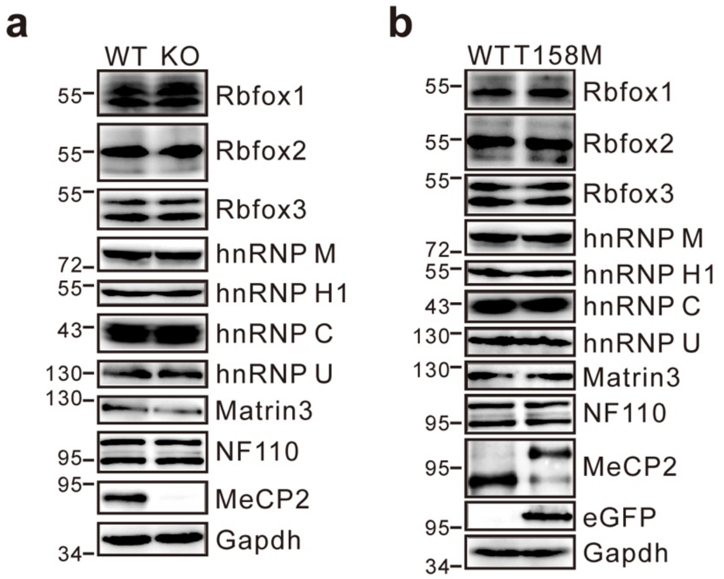

Supplementary Fig. 7 The expression of Rbfox proteins and LASR components in the cerebral cortex of WT, MeCP2 KO and T158M mice. a, **b** Western blot analyses of Rbfox proteins and LASR components in the cerebral cortex of MeCP2 KO (**a**), T158M (**b**) mice, and their WT littermates. Source data are provided as a Source Data file.

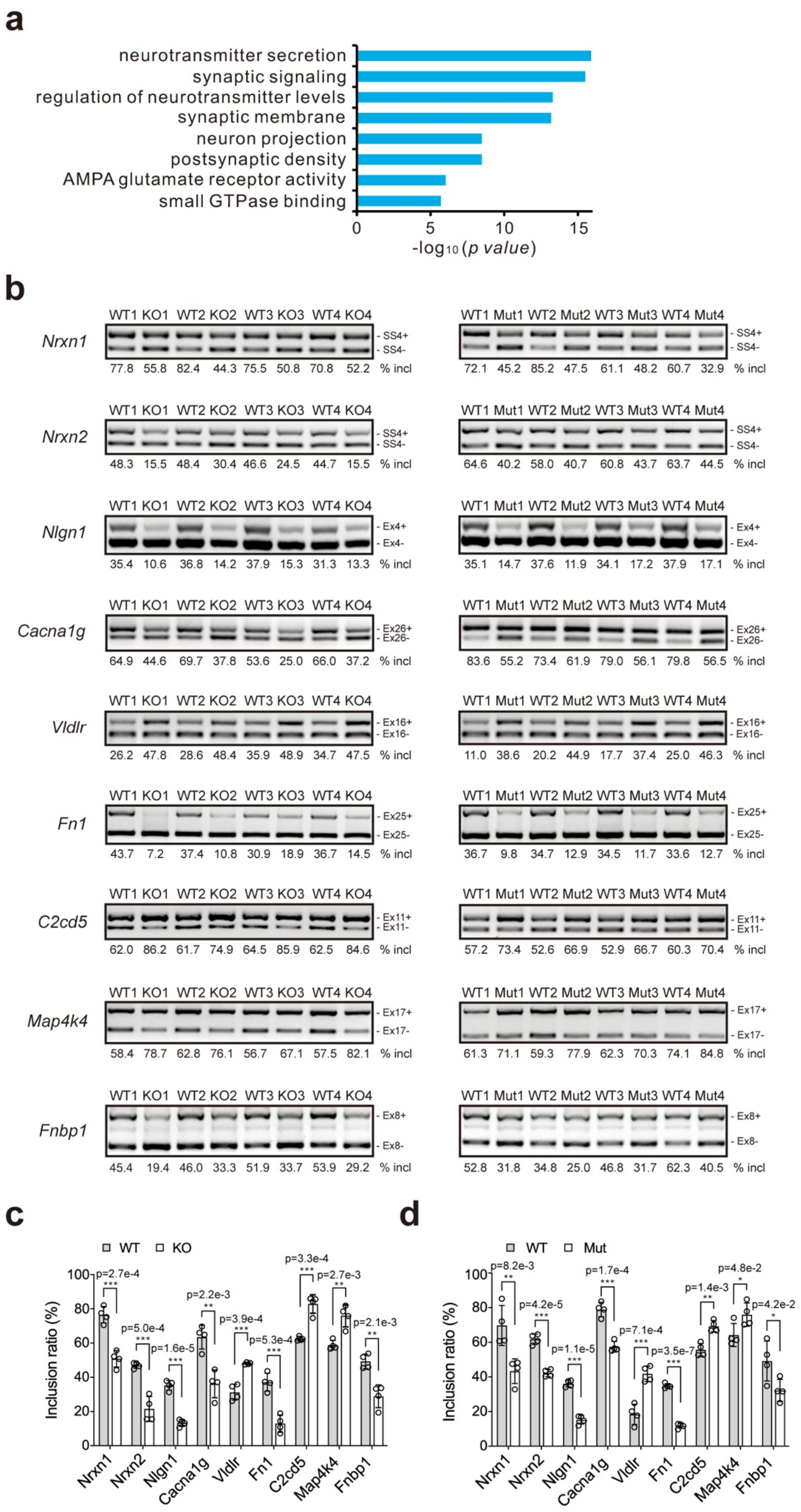
a
neurotransmitter secretion
synaptic signaling
regulation of neurotransmitter levels
synaptic membrane
neuron projection
postsynaptic density
AMPA glutamate receptor activity
small GTPase binding
-log10 (p value)
b
Nrxn1
Nrxn2
Nlgn1
Cacna1g
Vldlr
Fn1
C2cd5
Map4k4
Fnbp1
WT1 KO1 WT2 KO2 WT3 KO3 WT4 KO4
WT1 Mut1 WT2 Mut2 WT3 Mut3 WT4 Mut4
% incl
c
d
WT
KO
Mut
Inclusion ratio (%)

Supplementary Fig. 8 Splicing changes detected in both MeCP2 KO and T158M mice. a Bar plots for GO categories of genes that undergo splicing changes in the cerebral cortex of both MeCP2 KO and T158M mice. **b** Representative RT-PCR results of splicing targets detected in the cerebral cortex of WT, MeCP2 KO and T158M mice. The percentages of exon inclusion were quantitated by GelAnalyzer and shown below the gel images. **c**, **d** Quantitation of changes in exon inclusion ratios shown in **b.** Error bars represent standard deviations (n=4 biological replicates; * $p<0.05$, ** $p<0.01$, *** $p<0.005$, Two-sided Student's t-test). Source data are provided as a Source Data file.

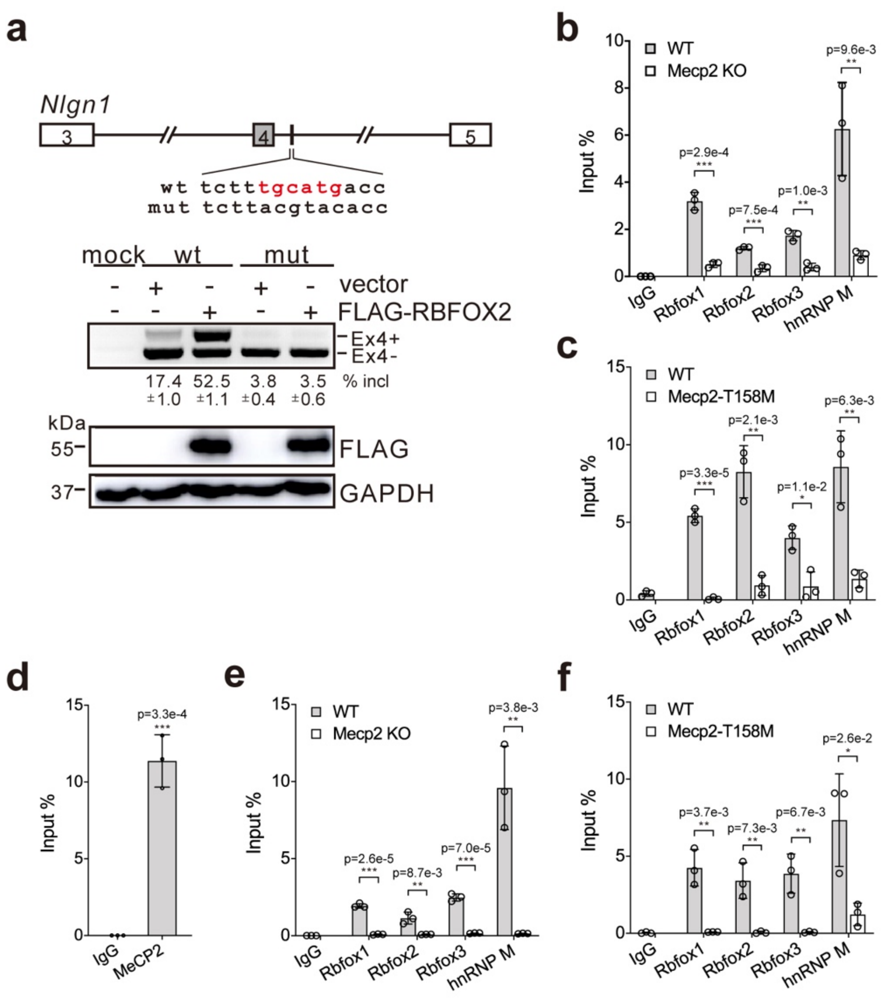

Supplementary Fig. 9 Regulation of *Nlgn1* exon 4 splicing in MeCP2 KO and T158M mice. a Schematic representation of *Nlgn1* minigenes and the *in vivo* splicing of *Nlgn1* minigenes in HEK293T cells. **b**, **c** CLIP-RT-qPCR analyses of Rbfox proteins and hnRNP M binding to *Nlgn1* pre-mRNAs in MeCP2 KO (**b**) or T158M (**c**) mice compared to WT mice. **d** ChIP-qPCR analysis of MeCP2 binding to *Nlgn1* genomic locus. **e**, **f** ChIP-qPCR analyses of Rbfox proteins and hnRNP M associating with *Nlgn1* genomic locus in MeCP2 KO (**e**) or T158M (**f**) mice compared to WT mice. Error bars in **b-f** represent standard deviations (n=3 biologically independent experiments; * p<0.05, ** p<0.01, *** p<0.005, Two-sided Student's t-test). Source data are provided as a Source Data file.

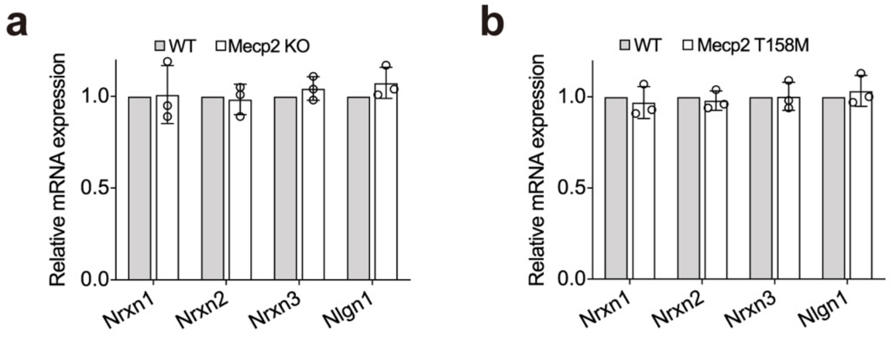

Supplementary Fig. 10 Expression levels of total *Nrxn1/2/3* and *Nlgn1* mRNAs in WT and MeCP2 mutant mice. a, **b** RT-qPCR analyses of *Nrxn1/2/3* and *Nlgn1* mRNA in MeCP2 KO (**a**) or T158M (**b**) mice compared to WT mice. Error bars represent standard deviations (n=3 biologically independent experiments; no significant changes were detected by Two-sided Student's t-test). Source data are provided as a Source Data file.

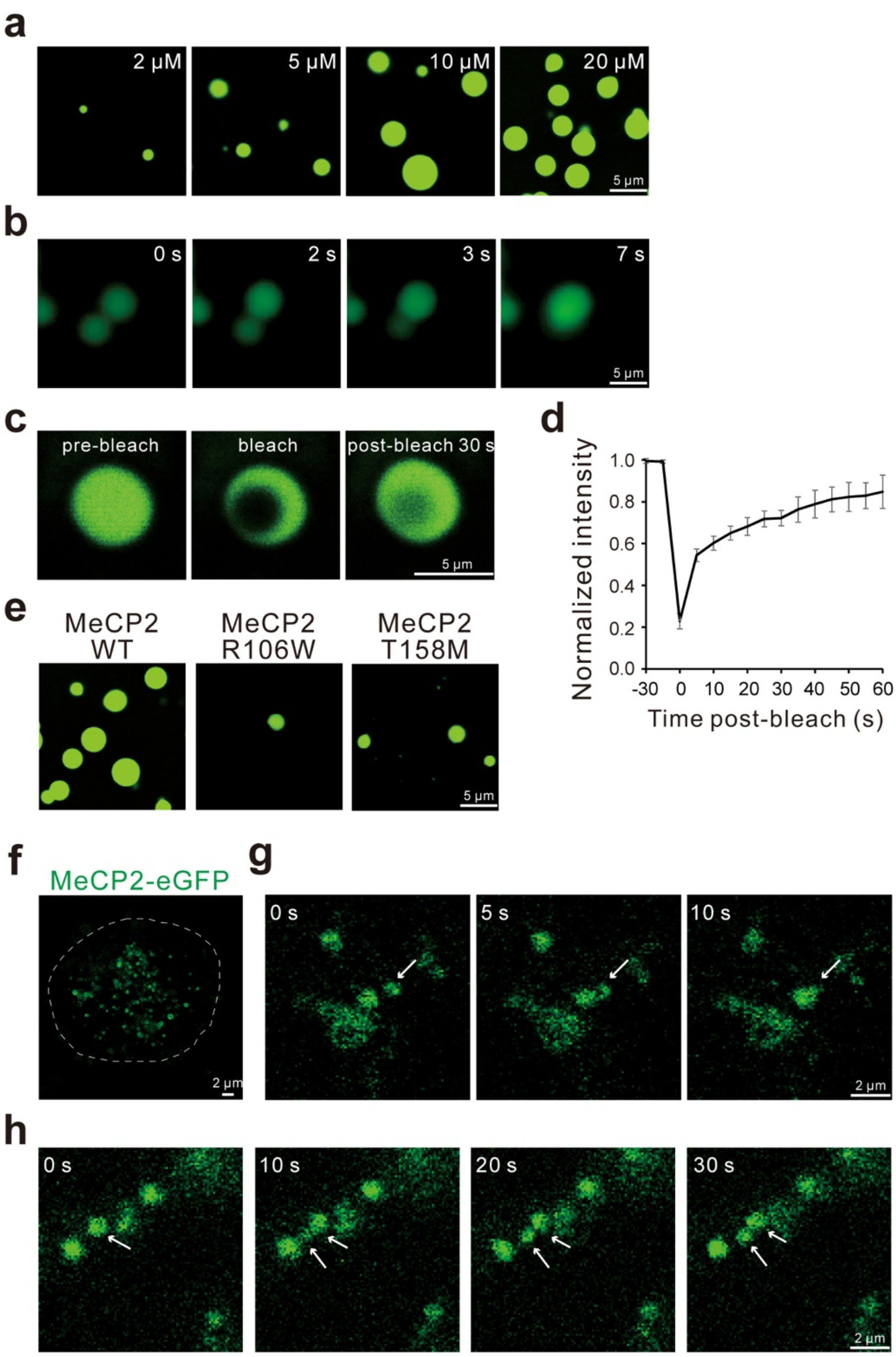

Supplementary Fig. 11 MeCP2 undergoes phase condensation both *in vitro* and *in vivo*. **a** Fluorescence images of MeCP2 with indicated concentrations at 37.5 mM salt condition. Scale bar: 5 μm. **b** MeCP2 droplets undergo liquid-like fusion. Scale bar: 5 μm. **c** FRAP analysis of MeCP2 droplets. Scale bar: 5 μm. **d** Quantitation of data shown in (**c**). Error bars represent standard deviations (n=12 cells). **e** Fluorescence images of droplets formed by 20 μM WT, R106W, and T158M MeCP2 at 37.5 mM salt condition. Scale bar:

5 μm. **f** Live cell image of MeCP2-eGFP fusion protein expressed in HEK293T eGFP tag knock-in cells. The nucleus is outlined by dotted lines. Scale bar: 2 μm. **g**, **h** MeCP2-eGFP protein undergoes liquid-like fusion (**g**) and fission (**h**). Scale bar: 2 μm. Source data are provided as a Source Data file.

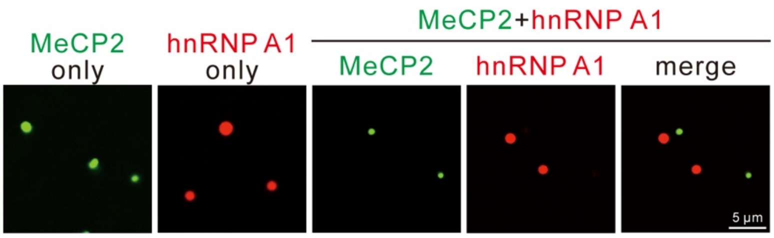

Supplementary Fig. 12 MeCP2 does not co-phase separate with hnRNP A1. Fluorescence images of droplets formed by MeCP2 and hnRNP A1 at 100 mM salt condition. Scale bar: 5 μm.

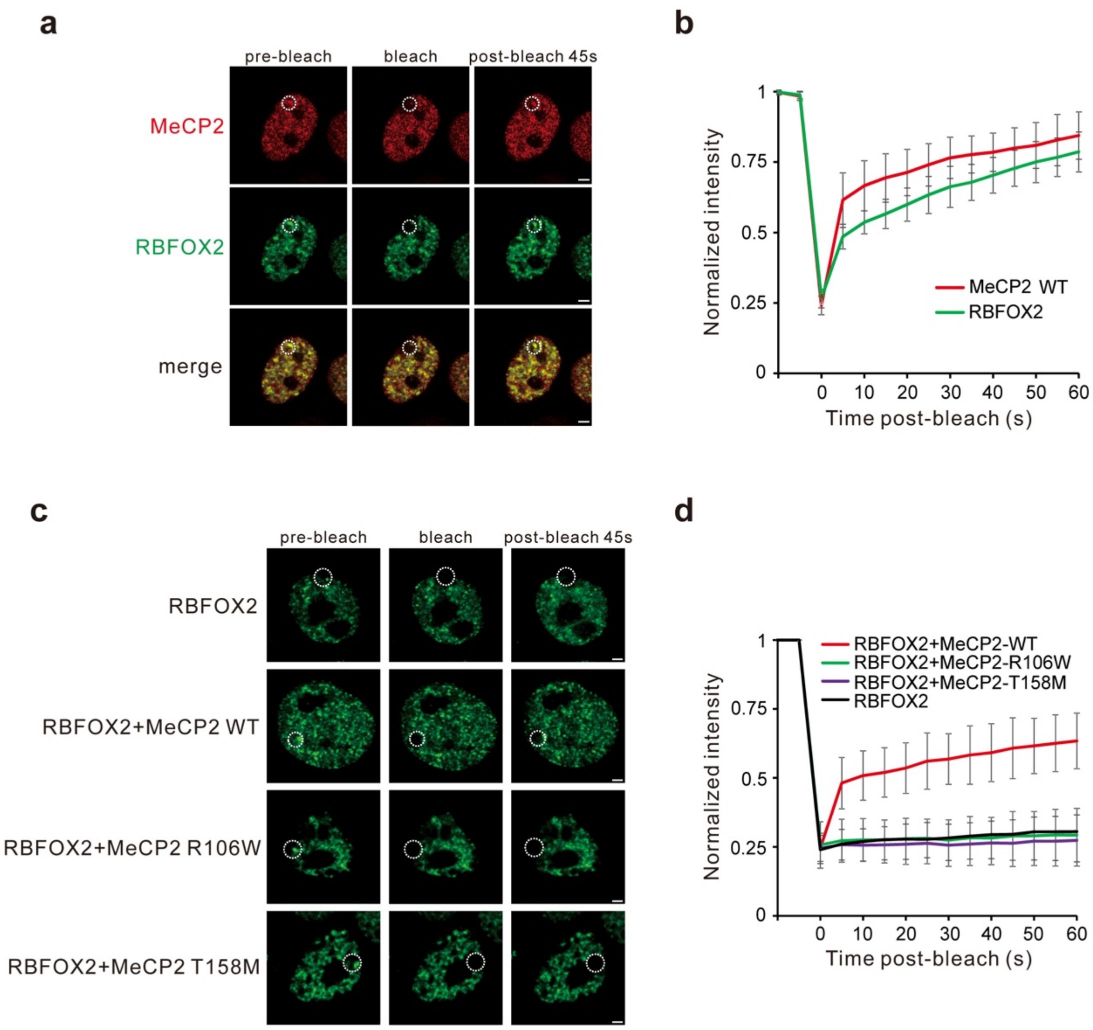

Supplementary Fig. 13 WT MeCP2, but not MeCP2 disease mutants, together with RBFOX2 forms condensates with liquid-like property. a Representative images showing FRAP analyses of MeCP2 and RBFOX2 proteins in WT HEK293T live cells. Scale bar: 2 μm. **b** Quantitation of data shown in (**a**). Error bars represent standard deviations (n=28 cells). **c** Representative images showing FRAP analyses of RBFOX2 alone or together with WT or mutant MeCP2 proteins in MeCP2 KO HEK293T live cells. Scale bar: 2 μm. **d** Quantitation of data shown in (**c**). Error bars represent standard deviations (n=30 cells). Source data are provided as a Source Data file.

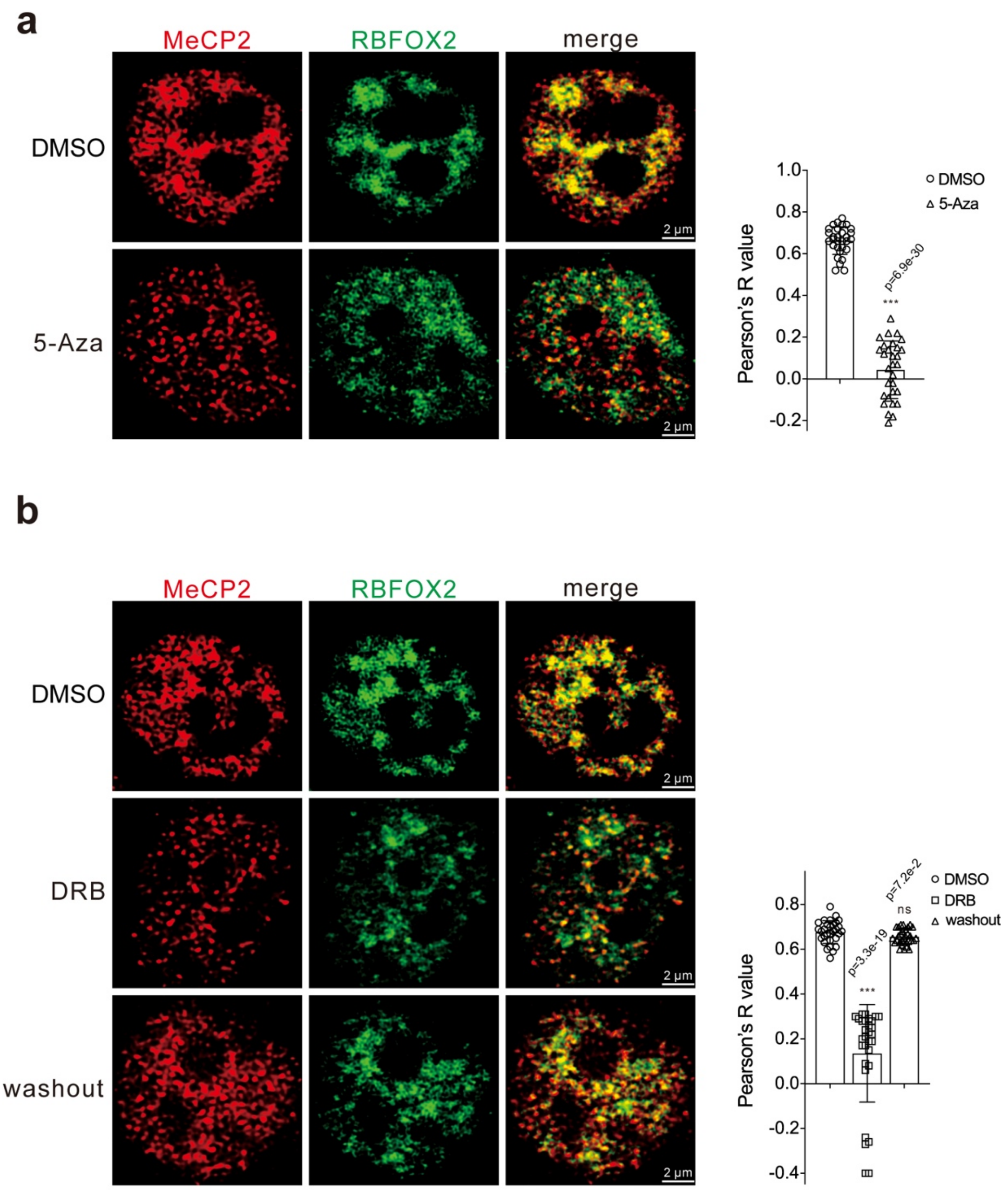

Supplementary Fig. 14 The co-localization of MeCP2 and RBFOX2 depends on DNA methylation and nascent RNA synthesis. a Representative immunofluorescence images of endogenous MeCP2 and Rbfox2 in HEK293T cells treated with DMSO or 5-Aza. **b** Representative immunofluorescence images of endogenous MeCP2 and Rbfox2 in HEK293T cells treated with DMSO, DRB, or DRB followed by washout. Pearson's R value analyses are shown on the right. Error bars represent standard deviations

(n=30 cells; *** $p<0.001$, Two-sided Student's t-test). Scale bar, 2 μm. Source data are provided as a Source Data file.

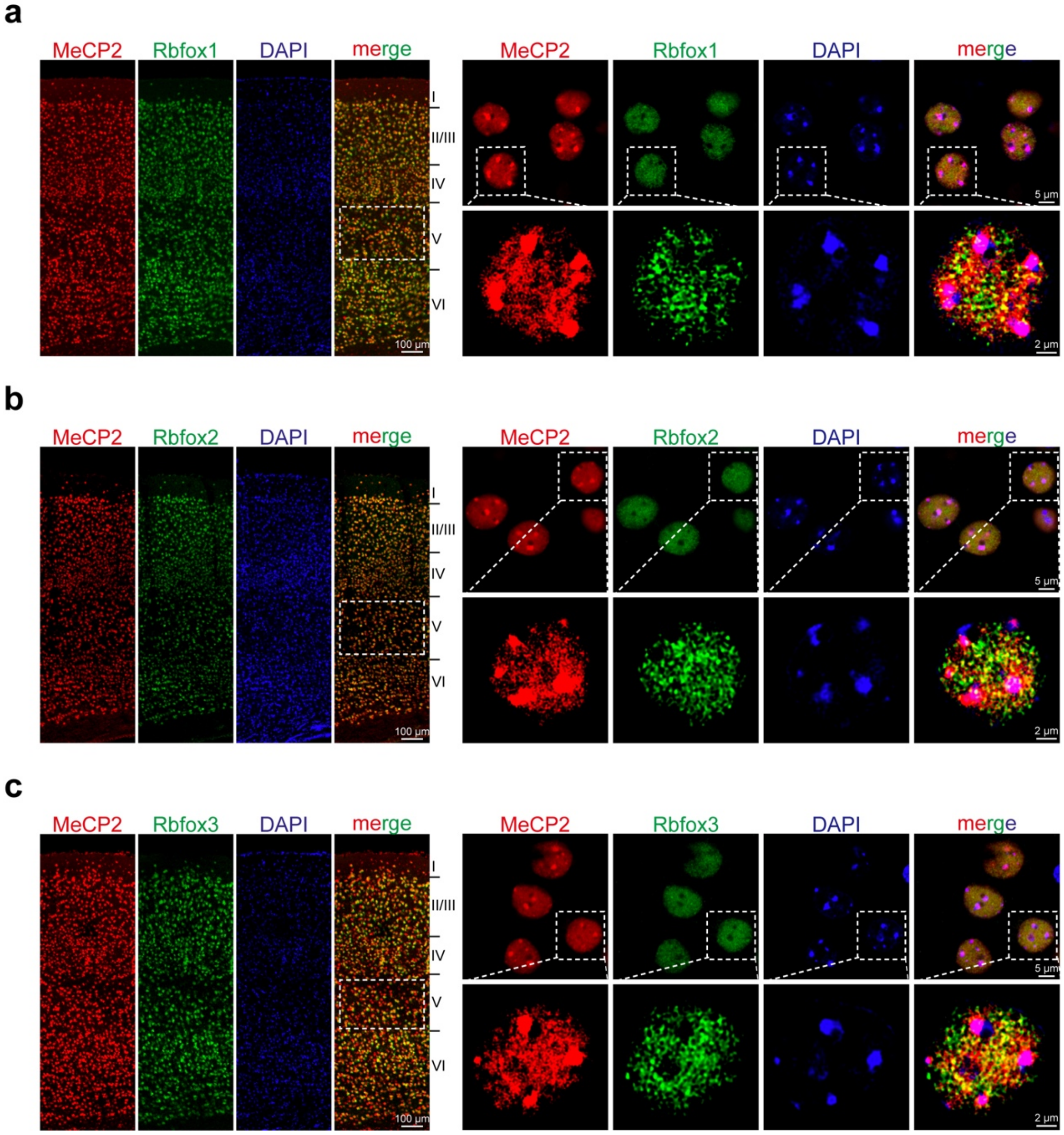

Supplementary Fig. 15 Localization of MeCP2 and Rbfox proteins in cortical neurons. a, b, c Representative confocal images of MeCP2, Rbfox1 (**a**), Rbfox2 (**b**) and Rbfox3 (**c**) in coronal sections of WT mice. Left panels: White dashed rectangles indicate cortical layer V. Scale bar, 100 μm. Right top panels: Scale bar, 5 μm. Right bottom panels: Scale bar, 2 μm.

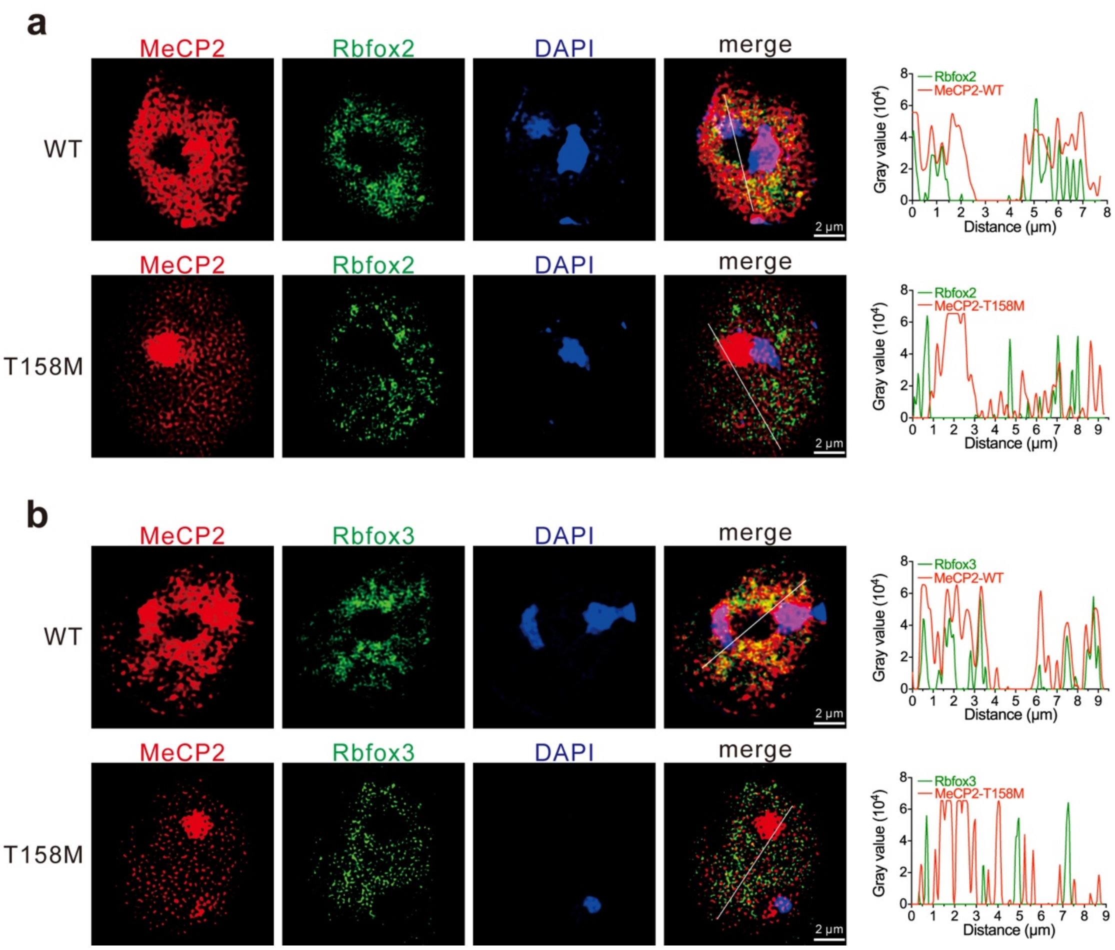

Supplementary Fig. 16 MeCP2 forms more condensates with Rbfox2 or Rbfox3 in WT mice than in MeCP2 T158M mice. a, **b** Representative immunofluorescence images of endogenous MeCP2 and Rbfox2 (**a**) or MeCP2 and Rbfox3 (**b**) in the brains of WT or MeCP2 T158M mice. Line scan graphs are shown on the right. Scale bar, 2 μm. Source data are provided as a Source Data file.

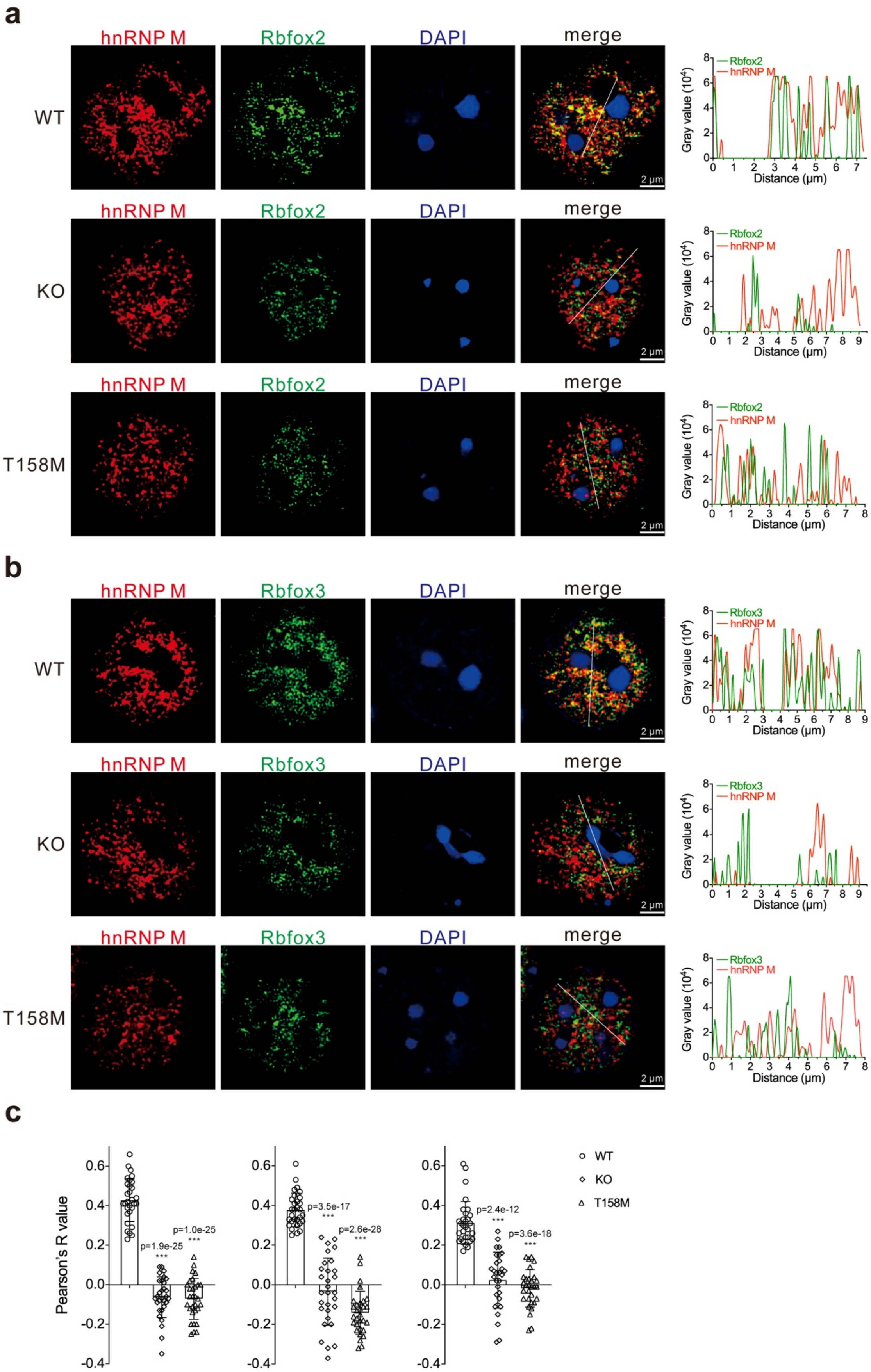
a
hnRNP M
Rbfox2
DAPI
merge
WT
KO
T158M
2 μm
Gray value (10⁴)
Distance (μm)
b
Rbfox3
c
Pearson's R value
p=1.9e-25
p=1.0e-25
p=3.5e-17
p=2.6e-28
p=2.4e-12
p=3.6e-18
***

Supplementary Fig. 17 Rbfox2 or Rbfox3 shows much less co-localization with LASR component, hnRNP M, in MeCP2 KO or T158M mice than in WT mice. a,**b** Representative immunofluorescence images of endogenous hnRNP M and Rbfox2 (**a**) or hnRNP M and Rbfox3 (**b**) in the brains of WT, MeCP2 KO or T158M mice. Scale bar, 2 μm. Line scan graphs are shown on the right. **c** Summary of Pearson's R values for hnRNP M and Rbfox1/2/3 in WT, MeCP2 KO and T158M mice. Error bars represent standard deviations (n=30 cells; *** $p<0.001$, Two-sided Student's t-test). Source data are provided as a Source Data file.

**Supplementary Table 1 Sequences of oligonucleotides used in this study**	
oligonucleotide name	oligonucleotide sequences (5'-3')
MeCP2-EcoRI-F	TTAAAGAATTCAGTAGCTGGGATGT
MeCP2-XbaI-R	TTAAATCTAGATCAGCTAACTCTCTC
MeCP2 ΔN-EcoRI-F	TTAAAGAATTCATCTGCCTCCCCCAAACAG
MeCP2 1-322-XbaI-R	TTAAATCTAGATTAGGGCTTCACCACTTCCTTG
MeCP2-ΔMBD-F	GAAGCTTCTAGCCCCTCCCGGCGAG
MeCP2-ΔMBD-R	GGAGGGGCTAGAAGCTTCCGGCACAGC
MeCP2-ΔID-F	GGGAGAGGGCAGGTGAAAAGGGTCCTGG
MeCP2-ΔID-R	TTTCACCTGCCCTCTCCCAGTTACCGT
MeCP2-ΔTRD-F	GTCAGAGGGTACGGTCAGCATCGAGGTC
MeCP2-ΔTRD-R	CTGACCGTACCCTCTGACGTGGCCG
MeCP2-R106W-F	GGCTGGACATGGAAGCTTAAGCAAAGGAAAT
MeCP2-R106W-R	TAAGCTTCCATGTCCAGCCTTCAGGCAG
MeCP2-T158M-F	TGACTTCATGGTAACTGGGAGAGGGA
MeCP2-T158M-R	CCCAGTTACCATGAAGTCAAAATCATTAGGGTC
Red-MeCP2-EcoRI-F	TTAAAGAATTCATGGTAGCTGGGAT
Red-MeCP2-BamHI-R	TTAAAGGATCCGCGCTAACTCTCTCGGTCACG
RBFOX2-HindIII-F	TTAAAAAGCTTATGGGATCCATGCA
RBFOX2-XhoI-R	TTAAACTCGAGTTAGACCTTCCGCTTCTTCTTTGGTT TATCGTCATCGTCTTTGTAG
Rbfox2-EcoRI-F	TCGAGCTCAAGCTTCGAATTCTGAGAAAAAGAAAAT GGTAACTC
Rbfox2-BamHI-R	TTATCTAGATCCGGTGGATCCTCAGTAGGGGGCAAA TCGGCT
sgRBFOX2_1-1	CACCTTGGGTCTCCCTTTAGTTCC
sgRBFOX2_1-2	AAACGGAACTAAAGGGAGACCCAA
sgRBFOX2_2-1	CACCGACGTGAGACCCCTGCAAAT
sgRBFOX2_2-2	AAACATTTGCAGGGGTCTCACGTC
sgMeCP2_1-1	CACCGTTGATTGCGTACTTCGAAA
sgMeCP2_1-2	AAACTTTCGAAGTACGCAATCAAC
sgMeCP2_2-1	CACCGTCTTCTATCCGATCTGTGC
sgMeCP2_2-2	AAACGCACAGATCGGATAGAAGAC
Rbfox2-KI-F	TTGACATGCACTGATCATCC
Rbfox2-KI-NLS-R	GACCTTCCGCTTCTTCTTTG
Rbfox2-KI-NLS-F	CAAAGAAGAAGCGGAAGGTCTGAAGTGACGTGAGA CCCC
Rbfox2-KI-R	CCTACCTTGATAACATAGAAAA
RBFOX2-KI-Test-F	AGGCCCCTAGAAATTGTCTGC
RBFOX2-KI-Test-R	ATTCGTGCGCTTCTGAGTGA
MeCP2-EGFP-KI-sg_1	CACCGAGAGTTAGCTGACTTTACACGG
MeCP2-EGFP-KI-sg_2	AAACCCGTGTAAAGTCAGCTAACTCTC
MeCP2-EGFP-KI-left-F	GGCAGCCAGGCAGTGTGA
MeCP2-EGFP-KI-left-R	CCTTGCTCACGCTAACTCTCTCGGTCACG
MeCP2-EGFP-KI-F	AGAGTTAGCGTGAGCAAGGGCGAGGAG

MeCP2-EGFP-KI-R	TGTAAAGTCACTTGTACAGCTCGTCCATG
MeCP2-EGFP-KI-right-F	GAGCTGTACAAGTGACTTTACACGGAGCGGA
MeCP2-EGFP-KI-right-R	TTTAAAACAAGCGCAGGTATAT
RBFOX2-mCherry-KI-sg_1	CACCTCTCACGTCACTTCAGTAGGGGG
RBFOX2-mCherry-KI-sg_2	AAACCCCCCTACTGAAGTGACGTGAGA
RBFOX2-mCherry-KI-left-F	CAGTGACACCTCTGCCAG
RBFOX2-mCherry-KI-left-R	CCTTGCTCACGTAGGGGGCAAATCGGCT
RBFOX2-mCherry-KI-F	CCCCCTACGTGAGCAAGGGCGAGGAG
RBFOX2-mCherry-KI-R	CGTCACTTCACTTGTACAGCTCGTCCATG
RBFOX2-mCherry-KI-right-F	GCTGTACAAGTGAAGTGACGTGAGACCCC
RBFOX2-mCherry-KI-right-R	TGGAAGATGAAAATTCCTATGG
MeCP2-EGFP-KI-T-F	GCAAAGAGGAGAAGATGCCC
MeCP2-EGFP-KI-T-R	GAAGCACTGCACGCCGTA
RBFOX2-mCherry-KI-T-F	ATGTGTTGGGCTCTGGTCTG
RBFOX2-mCherry-KI-T-R	ATTTGCAGGGGTCTCACGTC
MeCP2-BamHI-F	TTAAAGGATCCCCATGGTAGCTGG
MeCP2-His-XhoI-R	TTAAACTCGAGTCAGTGGTGGTGGTGGTGGCTA ACTCTCTCGGTCACG
pGEX-6P-MeCP2-BamHI-F	TTAAAGGATCCGTAGCTGGGATGTTAGGGC
MeCP2-mid-R	CCTTGCTCACGCTAACTCTCTCGGTCACG
EGFP-F	GAGAGTTAGCGTGAGCAAGGGCGAGGAG
EGFP-EcoRI-R	TTAAAGAATTCCTTGTACAGCTCGTCCATG
twin-strep-EcoRI-F	TTAAAGAATTC TGGAGCCACCCGCAGTTC
twin-strep-XhoI-R	TTAAACTCGAGTTATTTTTCGAACTG
MBD-BamHI-F	GATCGAAGGTCGTGGGATCCCCTCTGCCTCCCCCA AACAG
MBD-EcoRI-R	GCTCGAGTCGACCCGGGAATTCTTATCTCCCAGTTA CCGTGAAG
ID- BamHI-F	GATCGAAGGTCGTGGGATCCCCGGGAGCCCCTCCC GGCGA
ID-EcoRII-R	GCTCGAGTCGACCCGGGAATTCTTACACACCCTCT GACGTGGC
BamHI-Rbfox2-F	TTAAAGGATCCATGCAGAACGAGCCTCTGAC
Rbfox2-mCherry-R	CCTTGCTCACGTAGGGGGCAAATCGGCT
Rbfox2-mCherry-F	TGCCCCCTACGTGAGCAAGGGCGAGGAG
XhoI-mCherry-R	TTAAACTCGAGTTACTTGTACAGCTCGTCCATGCCG CCGGTGG
BamHI-hnRNPA1-F	TTAAAGGATCCTCTAAGTCAGAGTCTCCTAAA
hnRNPA1-Cherry-R	CCTTGCTCACAAATCTTCTGCCACTGCCA
hnRNPA1-mCherry-F	CAGAAGATTTGTGAGCAAGGGCGAGGAG
HindIII-Nlgn1-F	ACTATAGGGAGACCCAAGCTTATGGCACTTCCCAGA TGCA
Nlgn1-I3-R	GGAAATTAGGAGCCCATTTC
Nlgn1-I3-F	TGGGCTCCTAATTTCCCATGGTGTATTTTTAATACTG AAA

Nlgn1-I4-R	AAAAAAAAAAAGCCCCTCTTTT
Nlgn1-I4-F	GAGGGGCTTTTTTTTTTTTCCTTACTTAATAGTTCATG AAT
Nlgn1-XhoI-R	CCCTCTAGATGCATGCTCGAGCAAGTACCCCAAGC CGATA
Nlgn1-mut-R	GTACGTAAGAGATTTGGTGGGAGAAAA
Nlgn1-mut-F	CCAAATCTCTTACGTACACCAGAGCCGCAATCACTT
HindIII-Nrxn3-F	ACTATAGGGAGACCCAAGCTTGAACAAGGGAAAATT GGAGT
Nrxn3-I16-R	AGAAACTTTAGGATATTTATCTTC
Nrxn3-I16-F	ATAAATATCCTAAAGTTTCTGAAAGATTAGATATTGAG CCTC
Nrxn3-I16-GA-R	TAAAGTTTAATCAACAGCTTAAAGAACAATCTGA
Nrxn3-I16-GA-F	CTTTAAGCTGTTGATTAAACTTTAAAGGCAACACTG
Nrxn3-I17-R	GAGAGGTAATTTAAAACAAATTAA
Nrxn3-I17-F	TTGTTTTAAATTACCTCTCAGATGTCTTCCTCCTTTGA ATA
Nrxn3-XhoI-R	CCCTCTAGATGCATGCTCGAGCTGAATGCTGGCTGT AGAG
Nlgn1-WT-1	AATTCCACCAAATCTCTTTGCATGACCAGAGCCGCA
Nlgn1-WT-2	AGCTTGCGGCTCTGGTCATGCAAAGAGATTTGGTGG
Nlgn1-Mut-1	AATTCCACCAAATCTCTTACGTACACCAGAGCCGCA
Nlgn1-Mut-2	AGCTTGCGGCTCTGGTGTACGTAAGAGATTTGGTGG
BGH-R	TAGAAGGCACAGTCGAGG
Nrxn1-SE-F	TGATGGGAAATACCATGTAGTACG
Nrxn1-SE-R	CCCGCCAATTATTATGGTTG
Nrxn2-SE-F	GGCAAATATCACGTGGTGC
Nrxn2-SE-R	TTGAAGATGGTCAGCTGGC
Nrxn3-SE-F	TATTGGCACAGTTGACATCTCC
Nrxn3-SE-R	TGGGTGTTGAAGATGGTTAGC
Nlgn1-SE-F	GATTGCCTGAAGTTATGCTTCC
Nlgn1-SE-R	GTACACCATCACTGGTTTGGG
C2cd5-SE-F	GCACGCTCTGTGAAGCTTTT
C2cd5-SE-R	TGCTGGTTGACTCGCTGTAG
Cacna1g-SE-F	GGTGGAGAACTTCCATAAGTGC
Cacna1g-SE-R	GAGTAGTAGGGTTTGCACTGGG
Vldlr-SE-F	CCCAATGGGTACAATCTCGAAG
Vldlr-SE-R	TTTTGGGGGAACACTGACTTCT
Fn1-SE-F	GACAAGGAAAGTGCCCCTATC
Fn1-SE-R	TCTTCACGGGTGAGTAGCG
Map4k4-SE-F	CAGGCTAAGCAAACGGGCAG
Map4k4-SE-R	GGACAGGGGAACGAGAGGTC
Fnbp1-SE-F	CACACAGCCAATGAAACGCA
Fnbp1-SE-R	GGTGGGAAGTTGCTGAAGTCT
Nlgn1-F	AGTCAACTATCGGCTTGGGG

Nlgn1-R	CTCGCTGGTCCATCTTAGGG
Nlgn1-RIP-F	TCTTTGCATGACCAGAGCCG
Nlgn1-RIP-R	ACATCTGAATGGTTGGCATCA
Nrxn1-F	AGATGACATCCTTGTGGCCT
Nrxn1-R	TATGGTTCACGGCCACCTAC
Nrxn1-RIP-F	AATTTGAACATAACCAAGTAGT
Nrxn1-RIP-R	AACCGTTGGTACTTCTAACA
Nrxn2-F	GAGCTGTTGGATGGCTACCT
Nrxn2-R	GAAGAGGTCTCTCACACAGCC
Nrxn2-RIP-F	TTCAGGCCGTTTGTCTCTGG
Nrxn2-RIP-R	TTCTGAGCACAAGCTGAGGG
Nrxn3-F	CTGGCCAGTGAATGAGCACT
Nrxn3-R	CTCCTCTACGGGCCGGTTAT
Nrxn3-RIP-F	TATAACCGGCCCGTAGAGGA
Nrxn3-RIP-R	GCAGGTGGAAGCTGACACAA
Gapdh-F	CCTGCACCACCAACTGCTTA
Gapdh-R	GGACACATTGGGGGTAGGAAC
L3-linker	/5Phos/UGAGAUCGGAAGAGCGGUUAG/3Pmn/
Rtclip2.0	GGATCCTGAACCGCT
L01clip2.0	/5Phos/NNNNATCACGNNNNNAGATCGGAAGAGCGT CGTG/3ddC/
L02clip2.0	/5Phos/NNNNCGATGTNNNNNAGATCGGAAGAGCGT CGTG/3ddC/
L03clip2.0	/5Phos/NNNNTTAGGCNNNNNAGATCGGAAGAGCGT CGTG/3ddC/
L04clip2.0	/5Phos/NNNNTGACCANNNNNAGATCGGAAGAGCGT CGTG/3ddC/
L05clip2.0	/5Phos/NNNNACAGTGNNNNNAGATCGGAAGAGCGT CGTG/3ddC/
L06clip2.0	/5Phos/NNNNGCCAATNNNNNAGATCGGAAGAGCGT CGTG/3ddC/
L07clip2.0	/5Phos/NNNNCAGATCNNNNNAGATCGGAAGAGCGT CGTG/3ddC/
P5Solexa_s	ACACGACGCTCTTCCGATCT
P3Solexa_s	CTGAACCGCTCTTCCGATCT
P5Solexa	AATGATACGGCGACCACCGAGATCTACACTCTTCCC TACACGACGCTCTTCCGATCT
P3Solexa	CAAGCAGAAGACGGCATACGAGATCGGTCTCGGCA TTCCTGCTGAACCGCTCTTCCGATCT
ssDNA-oligo1	5’-Biotin-TAGAAGAATTC (m5C) GTTCCAG-3’
ssDNA-oligo2	5’-CTGGAA (m5C) GGAATTCTTCTA-3’
