# Supplementary material for: Rett syndrome linked to defects in forming the MeCP2/Rbfox/LASR complex in mouse models
Source: Nat Commun. 2021 Oct 1;12:5767. doi: 10.1038/s41467-021-26084-3 (PMC8486766; doi:10.1038/s41467-021-26084-3)
Supplement: Supplementary file 7 — Description of additional supplementary files [file 41467_2021_26084_MOESM7_ESM.docx]

Description of additional supplementary files

Title: Supplementary Data 1

Description: Alternative splicing changes overlapped between MeCP2 KO and RBFOX2 ΔCTD KI HEK293T cells

Title: Supplementary Data 2

Description: Genomic mapping of RBFOX2 binding sites in WT and MeCP2 KO HEK293T cells by iCLIP-seq

Title: Supplementary Data 3

Description: Alternative splicing changes between WT and MeCP2 KO mice

Title: Supplementary Data 4

Description: Alternative splicing changes between WT and MeCP2 T158M mice

Title: Supplementary Data 5

Description: Overlapped splicing changes between MeCP2 KO and T158M mice
