# Supplementary material for: Rett syndrome linked to defects in forming the MeCP2/Rbfox/LASR complex in mouse models
Source: Nat Commun. 2021 Oct 1;12:5767. doi: 10.1038/s41467-021-26084-3 (PMC8486766; doi:10.1038/s41467-021-26084-3)
Supplement: Supplementary file 8 — Reporting Summary [file 41467_2021_26084_MOESM8_ESM.pdf]

nature research

Corresponding author(s): Ying Huang, Jingyi Hui

Last updated by author(s): Aug 28, 2021

Reporting Summary

Nature Research wishes to improve the reproducibility of the work that we publish. This form provides structure for consistency and transparency in reporting. For further information on Nature Research policies, see our Editorial Policies and the Editorial Policy Checklist.

Statistics

For all statistical analyses, confirm that the following items are present in the figure legend, table legend, main text, or Methods section.

n/a	Confirmed	
☐	☒	The exact sample size (n) for each experimental group/condition, given as a discrete number and unit of measurement
☐	☒	A statement on whether measurements were taken from distinct samples or whether the same sample was measured repeatedly
☐	☒	The statistical test(s) used AND whether they are one- or two-sided<br>*Only common tests should be described solely by name; describe more complex techniques in the Methods section.*
☒	☐	A description of all covariates tested
☐	☒	A description of any assumptions or corrections, such as tests of normality and adjustment for multiple comparisons
☐	☒	A full description of the statistical parameters including central tendency (e.g. means) or other basic estimates (e.g. regression coefficient) AND variation (e.g. standard deviation) or associated estimates of uncertainty (e.g. confidence intervals)
☐	☒	For null hypothesis testing, the test statistic (e.g. F, t, r) with confidence intervals, effect sizes, degrees of freedom and P value noted<br>*Give P values as exact values whenever suitable.*
☒	☐	For Bayesian analysis, information on the choice of priors and Markov chain Monte Carlo settings
☒	☐	For hierarchical and complex designs, identification of the appropriate level for tests and full reporting of outcomes
☐	☒	Estimates of effect sizes (e.g. Cohen's d, Pearson's r), indicating how they were calculated

Our web collection on statistics for biologists contains articles on many of the points above.

Software and code

Policy information about availability of computer code

Data collection	Immunofluorescence: Leica Application Suite X (v3.5.5.19976); Immunoblotting: Amersham Imager software 680 (v2.0.0); high throughput sequencing: Illumina HiSeq X Ten platform.
Data analysis	ImageJ (v2.0.0-rc-69/1.52p); Prism (v8 8.4.0); Excel (v16.38); GelAnalyzer (v19.1); Trimmomatic (v0.36); HISAT2 (v2.1.0); HTSeq (v0.9.1); rMATS (v4.0.2); PAIRADISE (v1.0); Flexbar (v3.5.0); STAR (v2.5.3a); UMI-tools (v0.5.1); BEDTools (v2.25.0); PureCLIP (v1.3.0); Bowtie2 (v2.3.2); SAMtools (v1.5); MACS (v1.4.2). The custom R script to perform the RNA map analysis is available at https://github.com/HJY-Lab/MeCP2_splicing.

For manuscripts utilizing custom algorithms or software that are central to the research but not yet described in published literature, software must be made available to editors and reviewers. We strongly encourage code deposition in a community repository (e.g. GitHub). See the Nature Research guidelines for submitting code & software for further information.

Data

Policy information about availability of data

All manuscripts must include a data availability statement. This statement should provide the following information, where applicable:

- Accession codes, unique identifiers, or web links for publicly available datasets
- A list of figures that have associated raw data
- A description of any restrictions on data availability

Sequencing data generated in this study are available in the Gene Expression Omnibus under accession number GSE142716. The mass spectrometry data are available at iProX database with the dataset identifier PXD021650 (www.iprox.org). Source data are provided with this paper.

Field-specific reporting

Please select the one below that is the best fit for your research. If you are not sure, read the appropriate sections before making your selection.

☒ Life sciences ☐ Behavioural & social sciences ☐ Ecological, evolutionary & environmental sciences

For a reference copy of the document with all sections, see nature.com/documents/nr-reporting-summary-flat.pdf

Life sciences study design

All studies must disclose on these points even when the disclosure is negative.

Sample size	The sample sizes were chosen based on previous studies using the same methodologies (Damianov et al., Cell, 165, 606-619, 2016; Han et al., Mol Cell, 65, 539-553, 2017) and accepted standards in the field.
Data exclusions	No data were excluded.
Replication	Only datasets with replicates were included in the study. The quality of replicates was assessed by PCA.
Randomization	All animals with a certain genotype were randomly selected.
Blinding	Blinding was not necessary since proper controls were included.

Reporting for specific materials, systems and methods

We require information from authors about some types of materials, experimental systems and methods used in many studies. Here, indicate whether each material, system or method listed is relevant to your study. If you are not sure if a list item applies to your research, read the appropriate section before selecting a response.

Materials & experimental systems

n/a	Involved in the study	
☐	☒	Antibodies
☐	☒	Eukaryotic cell lines
☒	☐	Palaeontology and archaeology
☐	☒	Animals and other organisms
☒	☐	Human research participants
☒	☐	Clinical data
☒	☐	Dual use research of concern

Methods

n/a	Involved in the study	
☐	☒	ChIP-seq
☒	☐	Flow cytometry
☒	☐	MRI-based neuroimaging

Antibodies

Antibodies used	The primary antibodies used in this study were anti-FLAG (F3165) from Sigma, anti-GAPDH (MM001) from ImB, anti-MeCP2 (C15410052) from Diagenode, anti-Rbfox1 (MABE159) and anti-Rbfox3 (MAB377) from Millipore, anti-Rbfox2 (A300-864A) and anti-Matrin3 (A300-591A) from Bethyl, anti-hnRNP M (sc-20001), and anti-hnRNP A1 (sc-32301) from Santa Cruz, anti-hnRNP C (A0057), anti-hnRNP H1 (A5924), anti-hnRNP U (A9907), anti-hnRNP F (A5505), anti-NF110 (A2496), and anti-hnRNP K (A1701) from Abclonal, anti-histone H3 (ab1791) and anti-DDX5 (ab21696) from Abcam, anti-GFP (66002-1-Ig) from Proteintech. The HRP-conjugated secondary antibodies were anti-mouse IgG (W4021) and anti-rabbit IgG (W4011) from Promega, conformation specific anti-rabbit IgG (3678) from Cell Signaling.
Validation	All the primary antibodies were validated for recognizing human or mouse proteins in immunoprecipitation, immunoblotting, or immunostaining assays by the manufactures including Sigma, Diagenode, Millipore, Bethyl, Santa Cruz, Abclonal, Abcam, Proteintech, and ImB. Statements regarding validation can be found at the manufactures' websites.

Eukaryotic cell lines

Policy information about cell lines

Cell line source(s)	HEK293T from ATCC.
Authentication	No further authentication was performed for commercially available cell lines.
Mycoplasma contamination	The cell line was tested negative for mycoplasma contamination with the Mycoplasma Detection Kit (Invivogen, USA).

Commonly misidentified lines (See ICLAC register)	None.

Animals and other organisms

Policy information about studies involving animals; ARRIVE guidelines recommended for reporting animal research

Laboratory animals	All mice used for experiments were male and female C57BL/6 mice. Brain tissues were obtained from 6-week-old male mice. Sources and housing conditions for all mice used in this study are described in the Methods.
Wild animals	None.
Field-collected samples	None.
Ethics oversight	All mouse experiments were approved by the Institutional Animal Care and Use Committee (IACUC) at the Institute of Biochemistry and Cell Biology, Chinese Academy of Sciences and conducted in accordance with the guidelines of IACUC.

Note that full information on the approval of the study protocol must also be provided in the manuscript.

ChIP-seq

Data deposition

☒ Confirm that both raw and final processed data have been deposited in a public database such as GEO.

☒ Confirm that you have deposited or provided access to graph files (e.g. BED files) for the called peaks.

Data access links *May remain private before publication.*	Sequencing data are available in the Gene Expression Omnibus under accession number GSE142716.
Files in database submission	GSM4557679_Input.bedgraph.gz; GSM4557680_IP.1.bedgraph.gz; GSM4557681_IP.2.bedgraph.gz
Genome browser session (e.g. UCSC)	http://www.genome.ucsc.edu/s/bioxfu/mecp2

Methodology

Replicates	duplicates
Sequencing depth	total number of reads (Input R1: 29339191; Input R2: 29339191; IP1 R1: 26382181; IP1 R2: 26382181; IP2 R1: 23479877; IP2 R2: 23479877)<br>uniquely mapped reads (Input: 38062048; IP1: 37602603; IP2: 34061565)<br>length of reads (150 bp), paired-end
Antibodies	Anti-MeCP2 (C15410052) from Diagenode
Peak calling parameters	command and parameters for read mapping:<br>bowtie2 -p 30 -x hg38 -1 read1.fastq.gz -2 read2.fastq.gz|samtools view -Sh -q 30 -F 4 -|grep -v 'XS:'|samtools view -Shub|samtools sort -o output.bam<br>command and parameters for peak calling:<br>macs14 -t treat.bam -c control.bam -g hs -p 1e-5 --nomodel
Data quality	There are 3677 and 6122 peaks were called with p-value less than 1e-5 and above 5-fold enrichment in two replicates, respectively.
Software	bowtie2, MACS, samtools, bedtools, ChIPseeker
